# Self-consistent signal transduction analysis for modeling context-specific signaling cascades and perturbations

**DOI:** 10.1038/s41540-024-00404-x

**Published:** 2024-07-19

**Authors:** John Cole

**Affiliations:** SimBioSys Inc., Champaign, IL USA

**Keywords:** Cancer, Regulatory networks

## Abstract

Biological signal transduction networks are central to information processing and regulation of gene expression across all domains of life. Dysregulation is known to cause a wide array of diseases, including cancers. Here I introduce self-consistent signal transduction analysis, which utilizes genome-scale -omics data (specifically transcriptomics and/or proteomics) in order to predict the flow of information through these networks in an individualized manner. I apply the method to the study of endocrine therapy in breast cancer patients, and show that drugs that inhibit estrogen receptor *α* elicit a wide array of antitumoral effects, and that their most clinically-impactful ones are through the modulation of proliferative signals that control the genes *GREB1, HK1, AKT1, MAPK1, AKT2*, and *NQO1*. This method offers researchers a valuable tool in understanding how and why dysregulation occurs, and how perturbations to the network (such as targeted therapies) effect the network itself, and ultimately patient outcomes.

## Introduction

Biological signal transduction networks (STNs) constitute the primary mechanism through which living cells sense their environment and adapt their behavior. Nearly every living thing—from the simplest bacterium swimming toward food, to a developing embryo—utilizes complex biochemical networks of interacting species to activate or inhibit different genes or cellular components. Despite their general robustness^1^, aberrant network behaviors can give rise to a variety of diseases including cancer.

A number of methods have been developed for modeling and simulating STNs, including coupled ordinary differential equations, partial differential equations, boolean or logic-based methods and their extensions (which treat elements of the network as though they are “on” of “off”), among many others^2–12^.

Constraint-based models, including flux balance analysis (FBA), leverage linear programming to study large reaction networks like metabolism (for an outstanding introduction to FBA, see ref. ^13^). FBA does not require detailed kinetic parametrization. Instead, modulation of constraints (e.g., limiting uptake, or preventing flux through enzyme-mediated reactions) is used to model environmental or biomolecular perturbations. Entire -omics data sets can be applied to model disease states, or specific cells—known broadly as context-specific FBA^14^. Despite their successes, constraint-based models incorporating signal transduction are rare, with notable applications including the ME model of O’Brien et al.^15^, the work of Vardi et al.^16^, and the lpNet models of Knapp et al. and Matos et al.^17,18^.

In this article I present a constraint-based method for modeling the human STN, dubbed self-consistent signal transduction analysis (SCSTA), and apply it to the study of estrogen receptor (ER*α*)-targeted endocrine therapy (ET) in breast cancer. En route, I will build a model of the human STN comprising over 200, 000 interactions, and employ transcriptomics data from The Cancer Genome Atlas (TCGA Research Network: https://www.cancer.gov/tcga) to impose constraints on gene expression and signal flux throughout the STN in order to model ET in nearly 1000 patients. The model predicts that ET alters gene expression in ways that decrease proliferation and cell cycling, and increase cell death, immune-related behaviors, stemness, and metastasis, with the changes in proliferation being significantly associated with overall survival (OS). The pathways that drive proliferation change center around the genes *GREB1, HK1, AKT1, MAPK1, AKT2*, and *NQO1*. Finally, I will directly compare predicted changes in gene expression with actual measured changes in patients that underwent ET, finding that the predictions are reasonably well-correlated with experiment.

## Results

### SCSTA

The main result of this work is the method for constructing and simulating an SCSTA model. Here I describe and discuss various design choices, as well as possible extensions or alterations (see Supplementary Section 1.1 for a concise description of the main assumptions and/or formalisms used).

#### Network construction and translation to a Linear Program

**Fig. 1:**
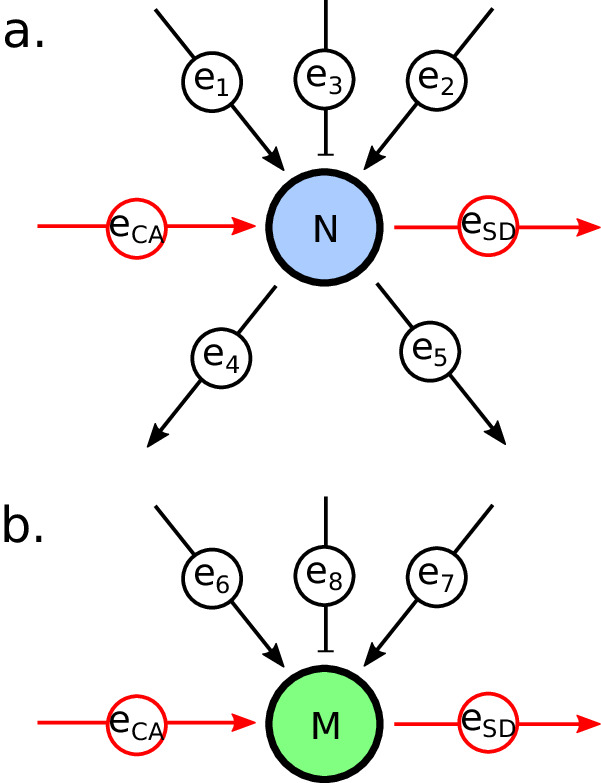
Cartoon representing typical nodes and edges. **a** Node corresponding to a typical gene product, with three parent edges (depicted as black arrows; two activating, one inhibiting), and two child edges (also shown in black; both activating). CA and SD edges are added, rendered in red. The value *N* represents the node’s gene product expression level. **b** Node corresponding to a typical gene with three parent edges (again, two activating and one inhibiting), along with CA and SD edges. The value *M* represents the node’s gene expression level. Note that no child edges are included; the total activation (less the SD) of this node should be proportional to *M*.

SCSTA requires some predetermined directed graph representing the signal transduction network of interest. The graph’s nodes represent biochemical species (small molecules, proteins, genes etc.), while edges represent interactions—a small molecule activating a protein, a protein phosphorylating another protein, or some transcription factor inhibiting some gene, etc. The edges are directed, meaning they have a “parent” node and a “child” node, and they may be either activating or inhibiting. Our SCSTA model is cast as a linear program (LP), and, similar to FBA, we assume it is at steady state. Edges correspond to variables in the LP, while nodes correspond to constraints. Figure 1a depicts a typical node; it has two activating and one inhibiting parent edges, and two child edges. We understand that the protein expression level of this node has some value *N*. We expect the node might display some level of constitutive activation, and it may also become spontaneously deactivated; accordingly we introduce constitutive activation (CA) and spontaneous deactivation (SD) edges (Fig. 1a). We assume the activating edges increase node activation, the inhibitory edges decrease activation, and complete activation occurs when all protein copies are activated, leading to the first constraint, *e*_CA_ + *e*_1_ + *e*_2_ − *e*_3_ ≤ *N*, or more generally:

1 $$\sum\limits_{{e}_{p}}{w}_{p}{e}_{p}\le N$$

where *e*_*p*_ represents each parent edge (including the CA edge), and *w*_*p*_ is some constant that, at the very least, encodes whether the edges activate or inhibit. Here I use *w*_*p*_ = 1 or −1 for activation or inhibition, although other values could be chosen (e.g., if a protein more readily interacts with some of its targets than others).

Activation “flows” out of the nodes via child edges, and we expect there could be some associated non-unity gain, *G* (e.g., if a protein activates multiple targets faster than it becomes deactivated). We require the total activation impinging on a node to be passed through to the child edges (including the SD edge), with the out-flowing activation bound by the product of *G* and the impinging activation. This results in the pair of constraints (1/*G*)(*e*_4_ + *e*_5_ + *e*_SD_) ≤ *e*_CA_ + *e*_1_ + *e*_2_ − *e*_3_ ≤ *e*_4_ + *e*_5_ + *e*_SD_, or more generally:

2 $$\begin{array}{l}\sum\limits_{{e}_{c}}{e}_{c}\ge \sum\limits_{{e}_{p}}{w}_{p}{e}_{p}\\ \sum\limits_{{e}_{c}}{e}_{c}\le G\sum\limits_{{e}_{p}}{w}_{p}{e}_{p}\end{array}$$

where *e*_*c*_ represents the *c*th child edge (including the SD edge). In this work, *G* was set conservatively at 2 (see Methods Section “Gain in SCSTA”).

Gene nodes (Fig. 1b) do not have child edges (save for an SD edge), but they still produce something important—transcripts. We assume the transcript expression level is proportional to the gene activation (less the SD edge), and that it is measured, denoted here as *M*. This gives the constraint (*e*_CA_ + *e*_6_ + *e*_7_ − *e*_8_ − *e*_SD_)*ξ* = *M*, or more generally:

3 $$\xi \left(-{e}_{{{{\rm{SD}}}}}+\sum\limits_{{e}_{p}}{w}_{p}{e}_{p}\right)=M$$

where *ξ* represents the proportionality between the gene’s activation and the transcripts produced (see Methods Section “Estimating ξ”).

Because paired transcriptomics and proteomics experiments are relatively rare, direct measurements of *N* and *M* for each gene are often unavailable. Here I assume that *N* and *M* are proportional to each other (i.e., there exists some *γ*_*i*_ such that *N*_*i*_ = *γ*_*i*_*M*_*i*_ for each transcript and protein), and estimate those proportionality constants utilizing data from ref. ^19^ (see Methods Section “Estimating γ”, and Supplementary Fig. 1).

Next, we consider the boundary conditions in our model, namely the small molecules and “parentless” nodes (those that have no impinging edges). Here I assume that small molecules are available in excess, and thus place no upper bound on the activation that can flow from them. I note that modulation of these bounds could be used to simulate different environmental conditions on the STN. The parentless nodes I assume are constitutively activated, and set the lower bound on each *e*_CA_ to the node’s corresponding *N*.

Finally, we consider the objective(s) for our LP. My goal is to find the “simplest” solution possible—that is the one that requires the least amount of pathway activity in order to give rise to the observed gene expression state. I think of this as an application of Occam’s razor; given a set of solutions all of which yield the observed gene expression state, I prefer the one with the lowest total activity. In constraint-based metabolic models, this type of optimality is often called the parsimonious solution. Unfortunately, this choice also imbues the solution with certain unintended biases (see Supplementary Section 1.2), including a tendency toward increased reliance on constitutive activation at or near the gene nodes. To circumvent this bias, I first introduce the objective:

4 $${c}_{1}=\sum\limits_{n}\frac{1}{{2}^{{d}_{n}}}\left({e}_{{{{\rm{CA}}}},n}+{e}_{{{{\rm{SD}}}},n}\right)$$

where *n* runs over all nodes, and *d*_*n*_ is the “distance” along the shortest path from node *n* to its nearest gene. Here, the CA and SD activations are increasingly more disfavored as the network is traversed toward the genes. By minimizing *c*_1_, subject to all other constraints, I find a solution that is intrinsically biased against large CA and SD values in general, and specifically biased against large CA and SD values at or close to the gene nodes. Once I know the minimal value that *c*_1_ can take, I stop using it as an objective and instead use it as a new constraint (that is, I bound from above the value *c*_1_ can take; in this work it was set to 1% larger than its minimal value). I then introduce the second objective:

5 $${c}_{2}=\sum\limits_{i}{e}_{i}$$

which represents the total activity flowing through the network. By minimizing *c*_2_, subject to all other constraints, including the newly-added one bounding the value of *c*_1_, I find a parsimonious solution without the aforementioned bias toward constitutive activation at or near the genes. I call this the “baseline” solution.

#### Minimally-perturbed solution

We want to perturb the network and predict its response. Unfortunately, many perturbations of interest will alter gene expression, and our model was constructed with a pre-defined gene expression state. Here I introduce the minimally-perturbed solution (MPS). We apply perturbations to the network (e.g., by altering constraints), remove the constraints on gene expression (Eq. (3)), use baseline values to impose upper bounds on the CA and SD edges, and then seek the solution that is most like the baseline solution. This is accomplished by introducing slack and surplus variables for each edge, and adding the constraints:

6 $${e}_{i}^{{{{\rm{MPS}}}}}+{e}_{i}^{{{{\rm{slack}}}}}-{e}_{i}^{{{{\rm{surplus}}}}}={e}_{i}^{{{{\rm{baseline}}}}}$$

where ${e}_{i}^{{{{\rm{baseline}}}}}$ and ${e}_{i}^{{{{\rm{MPS}}}}}$ represent *i*th edge values in the baseline and new (minimally) perturbed solutions, the later being found by minimizing:

7 $${c}_{3}=\sum\limits_{i}{e}_{i}^{{{{\rm{slack}}}}}+{e}_{i}^{{{{\rm{surplus}}}}}$$

This approach seeks the smallest change that can result from a given perturbation, and thus represents a conservative starting point for numerous lines of inquiry. Drug targets could be elucidated by systematically preventing activation of each node (mimicking a strong inhibitor), or populations of patients that respond to new or existing drugs may be elucidated through screening of their transcriptomes.

I note that the MPS described here is not “self-consistent” in the same way that the baseline solution is; it uses unperturbed node expression levels (*N* values) to predict perturbed *M* values. This can be rectified in several ways, some of which are detailed in Supplementary Section 1.3. In this article, the simpler approach outlined here is used.

### Predicting Pre- and Post-ET in TCGA-BRCA patients

From TCGA, I downloaded every RNAseq dataset associated with the TCGA-BRCA project, excluding patients for which either multiple or no transcriptomics datasets were available. For each patient (970 in total), I computed the baseline and MPS associated with the inhibition of *ERα* (see Methods Section “Modeling ET in Breast Cancer”). I then computed scores for several cancer-associated behaviors (proliferation, cell death, cell cycle, immune, stemness, and metastasis) based on the gene expression profiles of each patient’s pair of solutions, and SIGNOR’s phenotypes dataset^20^ (see Methods Section “Computing phenotype scores”).

**Fig. 2:**
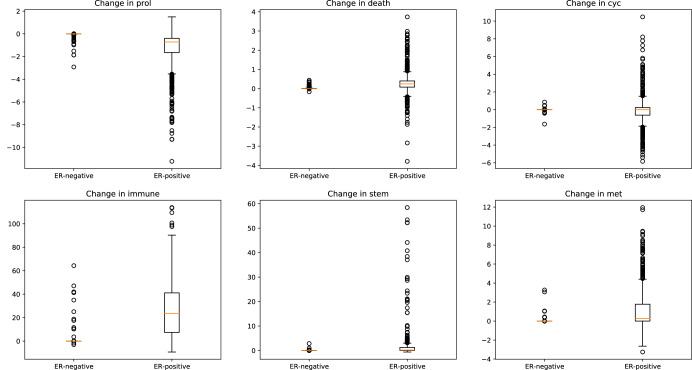
Boxplots of predicted changes in each behavior score under ET. For each behavior, patients are stratified by ER-positivity.

The ET-associated change in each score was computed (defined as pretreatment subtracted from post-treatment), and patients were categorized based on ER immunohistochemistry (IHC) status (when unavailable or equivocal, status was imputed based on transcriptomic data, see Methods Section “Imputation of feature values for TCGA patients”). As expected, we see much larger score changes associated with ER-positive than ER-negative patients, and for most ER-negative patients, no change was predicted (the inner-quartile range is zero, see Fig. 2; also see Supplementary Section 1.4 and accompanying Supplementary Tables 1--2, and Supplementary Fig. 2 for pretreatment scores stratified by ER-positivity).

Among ER-positive patients, score changes tended to be beneficial: the proliferation and cell cycle scores tend to decrease, while the cell death and immune scores tend to increase (Fig. 2 and Supplementary Fig. 3). The model also correctly predicts that the stemness and metastasis scores increase among ER-positive patients, possibly resulting from therapy driving these tumors into a more basal-like regulatory state. This is in keeping with the results of a 2011 study by Al Saleh et al., which found that estrogen receptor silencing in MCF7 cells drove a transition toward mesenchymal phenotype, increased metastatic propensity, and a shift toward basal cell markers^21^.

It has been reported that epithelial-mesenchymal transition (EMT, part of the stemness score) is associated with tamoxifen resistance^22,23^, and that this relationship is bidirectional and reversible—reductions in ER signaling (e.g., via targeted therapy) lead to increased EMT, and increases in EMT lead to decreased ER signaling^24^. In Supplementary Section 1.5, I explore this phenomenon further, recapitulating the results of^21^, and finding that the model does indeed predict greater (lesser) sensitivity to ET among patients with low (high) EMT scores, and that ER-expressing cell lines with high markers of EMT are predicted to be relatively insensitive to ET.

#### ET-associated changes in proliferation are associated with enhanced overall survival

Among ER-positive patients, adjuvant ET (extending between 5 and 10 years) has long been standard of care; this was true well before the TCGA-BRCA patients were studied and thus it is likely that many received ET (the TCGA has data on administered drugs like tamoxifen and fulvestrant, but the coverage is relatively low). I performed a multivariate Cox regression over all ER-positive patients (*N* = 749), including the change in each score (binarized at the median) along with several other covariates associated with survival (see Methods Section “Survival analysis”).

**Fig. 3:**
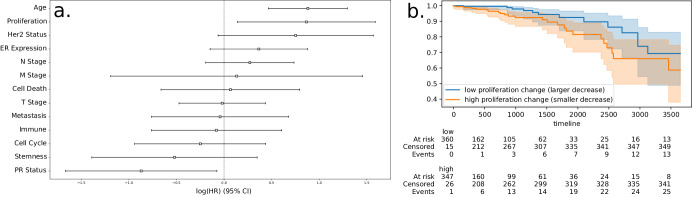
Survival analysis results. **a** Multivariate cox regression of the change in each behavior score, along with other meaningful covariates (bars indicate 95% confidence interval). For each of the six behavior scores (Proliferation, Cell Death, Cell Cycle, Immune, Stemness, and Metastasis), the difference in score value (post-ET minus pre-ET) was binarized at the median value over the population such that patients with a value below the median were assigned a value of 0, while those equal to or above the median were assigned a value of 1. Only the change in the proliferation score was significantly associated with OS. Because almost all patients experienced a decrease in proliferation under ET, the cohort with high values (i.e., small negative values) correspond to the smallest decreases and have worse OS, while those with low values (large negative values) correspond to the largest decreases and better OS. **b** Kaplan–Meier plot of ER-positive patients stratified by the median change in the proliferation score. Survival is shown in days. Width of colored regions surrounding both lines indicate 95% confidence intervals.

Only the change in proliferation was significantly associated with OS during the 10 years after diagnosis (see Fig. 3a; hazard ratio (HR) 2.38, log-likelihood ratio test *p* ≈ 0.02). Impressively, this was associated with a greater risk than T, N, and M stage, HER2 status, and *ESR1* expression. Only patient age and PR status had greater HRs. In a univariate analysis, the HR associated with proliferation change was 2.0 (*p* ≈ 0.04); an accompanying Kaplan–Meier plot is shown in Fig. 3b.

The change in proliferation score tended not to correlate with most other covariates (see Supplementary section 1.6 for details on this and other score changes, and Supplementary Fig. 4); for only two covariates—*ESR1* expression level, and the change in stemness score—did the magnitude of its Spearman correlation rise above 0.2 (−0.21 and −0.26, respectively). This indicates that: ET is predicted to be more effective in reducing cancer proliferation among patients with higher ER*α* expression (as should be expected); and that tumors predicted to undergo larger reductions in proliferation are also predicted to increase in stem-like behaviors.

The widely-used 21 gene Oncotype DX (R) Recurrence Score (RS) is heavily-weighted toward proliferative genes, supporting the notion that proliferation strongly influences risk of recurrence^25^. Although the RS was not designed for prognosticating OS, a recent study showed that when binarized at the median, it appears to show comparable performance to our computed proliferation score change^26^. I thus sought to determine whether the proliferation score change was correlated with its pretreatment value. I found their Pearson correlation coefficient to be *ρ* ≈ − 0.072, and observed no obvious trend (see Supplementary Fig. 5). I performed additional multivariate Cox regressions to determine if including the pretreatment score (binarized at its median) impacted the HR associated with the score change. In a bivariate analysis, both were found to have comparable HRs (2.20 for pretreatment score, and 2.16 for score change; *p*-values of 0.02; see Supplementary Fig. 6 for accompanying Kaplan–Meier plot), while in the more comprehensive analysis that included additional covariates, these HRs jumped to approximately 2.87 and 2.61 respectively (*p*-values of less than 0.005, and 0.01). All of this strongly supports the hypothesis that the changes in gene expression predicted by SCSTA, particularly among proliferative genes, offer insight into which patients benefit most from ET.

#### Pathways predicted to drive ET-associated changes in proliferation

I investigated which changes in pathway activation were most strongly associated with changes in the behavior scores. Because proliferation change is associated with OS, it will be presented here, while similar analyses of other score changes will be relegated to Supplementary Section 1.7 and accompanying Supplementary Fig. 7).

Over the entire ER-positive population, the fraction of total score change attributable to a given edge, denoted *ϕ*, was computed (see Methods Section “Determining drivers of changes in behavior scores”). I found a diverse set of edges with relatively small *ϕ*-values contributing toward reduced proliferation, with the largest seven accounting for just over half ( ≈ 53%; see Supplementary Tables 3–8 for *ϕ*-values associated with each score).

Of these seven driver edges, three were the result of ER*α*’s direct transcriptional regulation of *GREB1*, *AKT1*, and *AKT2* (the first, third, and fifth largest *ϕ*-values). *GREB1* is a known mediator of ET resistance^27,28^; it is strongly associated with ER-positive breast cancer, it is rapidly induced by estrogen, and that induction is critical for ER-associated proliferation^29,30^. *AKT1* and *2* are linchpin signal transducers in the PI3K/AKT/MTOR pathway, and represent central players in proliferative and anti-apoptotic signaling in many cancers, including breast cancer^31,32^.

**Fig. 4:**
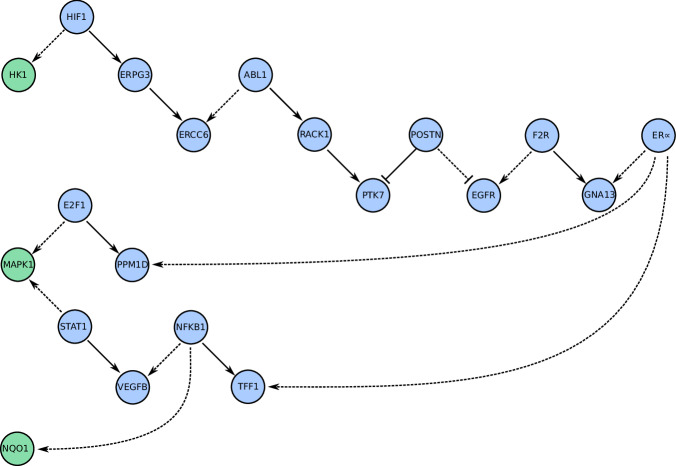
Pathway diagrams indicating how ET is predicted to decrease proliferation. For each MDP described in section “Pathways predicted to drive ET-associated changes in proliferation”, the affected gene is shown in green on the left, and the pathway proceeds to the right, terminating at ER*α*. In nearly all interactions, parent nodes are depicted above child nodes. The pathway regulating *HK1* (top) is the longest and is largely independent from the remaining three (bottom), which are tightly-coupled. The two pathways that regulate *MAPK1* diverge early, one of which also forms part of the pathway regulating *NQO1*. Edges are shown as solid or dashed arrows, indicating whether ET is predicted to increase or decrease the strength of the interaction.

The second largest *ϕ* corresponded to decreased activation of *HK1* by HIF1. HK1 is a key glycolytic enzyme, and although it has not been implicated directly in conferring sensitivity or resistance to ET, its isoform Hexokinase 2 has^33^. To understand how ER*α* signaling affects *HK1* expression, I computed the connecting pathway along which each edge shows the largest difference in activity between predicted pre- and post-ET solutions (see Methods section “Maximally-dysregulated pathways” for details). This “maximally-dysregulated pathway” (MDP) involved: the loss of activation of GNA13 by ER*α* being offset by F2R, at the cost of F2R’s normal activation of EGFR; the dysregulation of EGFR and PTK7 by POSTN, resulting in redirection of activity away from ERCC6; and partial offset of this loss of ERCC6 activity by the redirection of activity from the gene *HK1* (see Fig. 4, upper pathway). Several proteins involved in this pathway are associated with breast cancer prognosis (including ERCC6 and POSTN^34,35^) and/or sensitivity/resistance to endocrine or other chemotherapy (including EGFR, ABL1, GNA13, and PTK7^36–39^).

Transcriptional activation of *MAPK1* by both E2F1 and STAT1 decreased under ET (the fourth and sixth largest *ϕ* values). MAPK1 is a key transducer of proliferative and many other signals, while E2F1 and STAT1 are both critical regulators of proliferation and cell cycle control that have been implicated in endocrine resistance^40–45^. The MDP proceeding through E2F1 consists of only one additional step (see Fig. 4), while the pathway through the STAT1 involved the redirection of NFKB1’s activity from VEGFB to TFF1 to offset the later’s loss in activation by ER*α*, and subsequent redirection of STAT1’s activity from its downstream targets to VEGFB (see Fig. 4).

The seventh largest *ϕ* corresponded to decreased transcriptional activation of *NQO1* by NFKB1. *NQO1* encodes a key metabolic enzyme that has been associated with breast cancer progression^46^. The associated MDP again involves redirection of NFKB1’s activity to TFF1, but this time at the cost of NQO1’s transcriptional activation (see Fig. 4).

Two of these pathways involved the redirection of NFKB1’s activity toward TFF1. In addition to being a direct target of ER*α* within the model, TFF1 is also an indirect target through FOXA1. This suggests that those patients with relatively low levels of FOXA1 and TFF1 activation by ER*α*, along with relatively high levels of NFKB1 activation should see reduced clinical benefit from ET. This interpretation is in keeping with the results of a 2017 study by Yamaguchi et al., which showed that expression of ER*α*, FOXA1, and TFF1 were all significantly reduced in an MCF7-derived tamoxifen-resistant breast cancer cell line, and that the canonical and non-canonical NF*κ*B signaling pathways were significantly upregulated^47^.

A 2022 study by Xia et al. included serially-sampled transcriptomics data for 35 patients undergoing ET, 25 of which had pre-treatment data^48^. For each of these, the pretreatment and earliest post-treatment gene expression levels were extracted and the log_2_ fold changes (L2FCs) were computed for *GREB1, HK1, AKT1, MAPK1, AKT2*, and *NQO1*. One-sided t-tests revealed that *GREB1* and *HK1*—the two most important effectors of the proliferation score—were significantly down-regulated, and all but *AKT2* trended toward downregulation (*p*-values less than 0.5, see Table 1).

**Table 1:** One-sided *t*-test *p*-vales for the six genes most strongly associated with the change in proliferation score

Gene	*p*-value
*GREB1*	4.5 × 10^9^
*HK1*	0.013
*AKT1*	0.11
*MAPK1*	0.36
*AKT2*	0.54
*NQO1*	0.24

I note that considerable variability exists within the population. While patients’ predicted pathway activities generally led to stable or decreased proliferation, two outliers were observed for which the proliferation score was actually predicted to increase. In both cases, these increases were predominantly driven by the loss of transcriptional inhibition of *MYC*, resulting in increased expression, and, in turn, an increase in the proliferation score. Over then entire ER-positive cohort, increased *MYC* expression is among the top 20 drivers of proliferation score change (see Supplementary Table 3), but its impact is generally smaller than that of other drivers, including those described above. In the case of these two outliers, the more canonical pathways leading to decreased proliferation play a much smaller role (the ones terminating in *GREB1* and *HK1*, as examples, have *ϕ*-values of only 0.0044 and 0.0076, respectively), indicating that these patients may be intrinsically less sensitive to ET.

#### Direct comparison of SCSTA predictions with measured post-ET data

As a final test, I predicted the post-ET transcriptome for each of the 25 patients selected from ref. ^48^, and compared those results to the actual post-treatment transcriptomic measurements (see Methods Section “Modeling ET in 25 patients with pre- and post-treatment transcrip-tomes” for details).

**Fig. 5:**
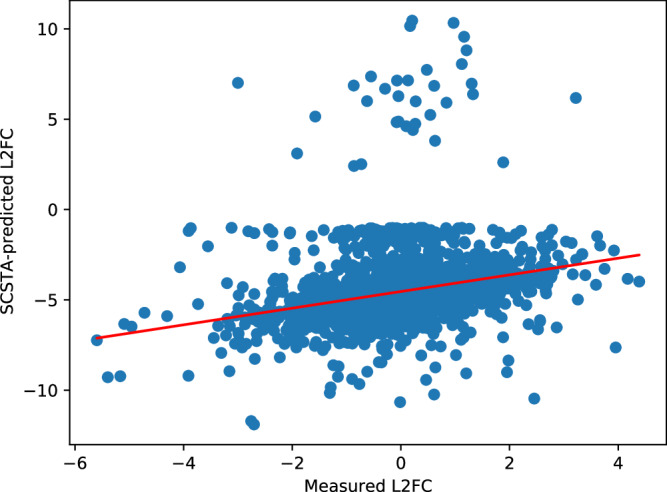
Scatter plot of SCSTA-predicted *v*s. measured L2FCs among the 3025 instances of modeled differential expression in the 25 patients from ref. ^48^. Blue dots indicate an instance (some gene in some patient) in which SCSTA predicted an absolute L2FC of greater than 1. The red line represents the linear least-squares best fit between predicted and experimental values. Overall, SCSTA tends to overpredict the degree of dysregulation under ET, and rarely predicts upregulation. Nevertheless, the predictions show a modest correlation with actual L2FC.

Over all genes in all patients, a total of 46,456 instances of apparent differential regulation (that is, a given gene in a given patient having an absolute L2FC of >1 between measured post- and pre-treatment expression levels) were observed. As expected, SCSTA was conservative in predicting differential expression, with only 3, 025 such instances found. It also tended to predict downregulation with much greater frequency than upregulation (2992 *vs*. 33 instances, respectively), and the magnitude of the predicted L2FCs was exaggerated relative to the measurements (see Fig. 5, and Supplementary Section 1.2 and accompanying Supplementary Fig. 8 for discussion of this effect). I note that the post-treatment measurements tended to be made weeks or months after treatment initiation, and may represent some evolution of the tumor cell population (e.g., expansion of less-sensitive clones) that could explain some of the noted discrepancies.

Among the predicted instances of differential expression, the Pearson and Spearman correlations with measured L2FCs were 0.27 and 0.42, respectively (indicating a modest, if nonlinear, monotonic relationship). As a comparator, I trained a multiple linear regressor (LR) to predict each of the 25 patient’s post-treatment transcriptomes from their pretreatment transcriptomes (see Methods Section “Linear Regression to predict changes in gene expression under ET”). The LR was less conservative in predicting differential regulation, with 55,138 instances overall, and, despite its use of actual post-treatment data during training, yielded both Pearson and Spearman correlation coefficients of 0.46, indicating greater linearity but only marginal improvement in Spearman correlation (see Supplementary Fig. 9).

## Discussion

In this article, I have presented SCSTA, a genome-scale constraint-based method for investigating STNs. The method’s most important conceptual leap lies in its self-consistency with respect to the transcriptional activation of genes, and their products’ engagement within the network. Specifically, SCSTA requires that the signal flux through the network reproduces the observed transcriptional profile of each patient, while simultaneously requiring that the gene products corresponding to that expression profile are capable of transmitting the required flux. I applied SCSTA to the study of one of the most widely-used classes of anti-cancer therapies in one of the most commonly-occurring cancer types, namely ET in breast cancer. I found that changes in the expression of proliferative genes, including *GREB1, HK1, AKT1, MAPK1, AKT2*, and *NQO1*, were associated with overall patient survival. In an independent dataset, all of these genes except *AKT2* showed evidence of downregulation under ET, and both *GREB1* and *HK1*—the two genes predicted to be most strongly associated with the change in proliferation—were significantly downregulated. Overall genes predicted to be dysregulated under ET, a modest correlation coefficient was observed.

SCSTA can be used in a variety of high-value applications, particularly in the quantitative systems pharmacology (QSP) sphere. Knock-out or knock-down studies of actual patients can be performed in order to: (1) elucidate new potential drug targets; (2) determine therapeutic levels of inhibition; (3) interrogate targeted multi-drug regimens; (4) predict patient populations that may benefit from a targeted therapy; and (5) elucidate potential mechanisms of action of drugs. The approach may also be integrated into other types of QSP models.

Although its current form admits steady-state approximations, other similar constraint-based models have been used in dynamical approaches. Mahadevan’s dynamic flux balance analysis (dFBA)^49^, for example, uses FBA coupled to a dynamic environment to simulate changes in metabolic behavior, while others, including myself, have extended these ideas to simulate microbial colonies and, more recently, solid tumors^50–52^. In its simplest form, the SCSTA model could be used as part of a pharmacodynamic description within a broader multicompartment pharmacokinetics/pharmacodynamics (PK/PD) simulation. A modeled drug could be dosed, and the concentration of the drug within the tumor compartment, for example, could be used to infer the degree of inhibition of the drug’s target. The SCSTA model could then be used to predict the effect on various readouts, proliferation or otherwise, at each simulation timepoint. The drugs that could be simulated include many already in the network (the set of small molecules extracted from OmniPath contains several drugs), but many more can be modeled. Indeed, the activity of any signaling molecule within the model may be modulated (e.g., partial or complete inhibition, or even increased activity) in order to simulate various existing, experimental, or hypothesized drugs.

There exist several opportunities for expansion and refinement of the approach. I note that the TCGA transcriptomics data used here represents bulk RNA samples, meaning multiple cell types (including infiltrating immune cells, fibroblasts, *etc*.) are present, to varying degrees, in each sample. While this naturally introduces some uncertainty when interpreting the results presented in this manuscript, it also presents an exciting opportunity for further development. In particular, Supplementary Section 1.8, and related Supplementary Fig. 10, describes one possible avenue through which bulk RNAseq may be deconvolved, and a compartmentalized SCSTA model representing multiple cell types interacting within a shared microenvironment may be constructed. Such an approach may be used to interrogate various forms of intercellular signaling, including potentially critical interactions between cancer cells and infiltrating lymphocytes. In a similar vein, single-cell RNAseq data could be used to characterize the heterogeneity in signaling and or drug sensitivity within a sample. This can be accomplished with a straightforward application of SCSTA directly to each cell sample. Such analyses could be used to predict if and why a given patient may develop resistance to a targeted therapy (e.g., if some sub-population of cells is intrinsically insensitive), or to design personalized multi-drug combinations targeting different subsets of cells. One could even envision combining a single-cell analysis with compartmentalized SCSTA described above; spatially-resolved RNAseq could be leveraged to create a representation of a microenvironment where each cell is its own compartment, and intercellular interactions may proceed among cells making direct contact with each other.

Much work is remains to be done. The development of carefully curated models of metabolism have taken decades, and refining the underlying STN used here in a similar manner will be invaluable. Moreover, many assumptions and approximations have been employed that could be refined, including the possible use of other optimality criteria (in light of the biases introduced by parsimony), better estimates of the weights in Eqs. (1)–(3) (perhaps based on high-throughput knockout experiments), or the inclusion of protein-specific estimates of signal gain (perhaps based on kinetics data), or the refinement of the weights in the behavior scores, to name just a few.

## Methods

### Gain in SCSTA

One of the parameters of the model presented in this work is the gain, *G*, which is intended to account for the possibility that proteins or other effectors may be able to interact with multiple targets before becoming deactivated. More conservative values (near 1) essentially require that activation be conserved as it is passed on through the network. Larger values enable small perturbations in upstream targets to have large downstream consequences, a feature of many biological networks, but they also somewhat decouple the activity flowing through the network from the expression levels of the nodes and the activity levels of upstream pathways (for example, a very large *G* could allow essentially arbitrarily-valued activation levels to appear at a given node despite that node’s parents’ activation levels being near zero). In principle, different gain parameters could be defined for each node in the network, but in this work, for simplicity, I used a single value for all nodes.

One way to roughly estimate the scale of *G* is by assuming that every node, when fully activated, might be able to fully activate each of its downstream targets. I considered the ratios of child node expression to parent node expression for each node in the STN. In order to do this, for each node I first selected the median protein expression value over all tissues and replicates in^19^. If a protein was not represented in the data, I used the product of the median *γ* value and the median transcript level for the gene encoding that protein, or failing that, the median protein expression level over all observations. Proteins were then redistributed to complexes when appropriate (see Methods Sections 4.3–4.4). Then for each node I computed the ratio of the sum of expression levels of all of the node’s targets to the node’s expression level, and then computed the median over all nodes, as:

8 $$G \sim {{{\rm{med}}}}\left[\frac{{\sum }_{t}{N}_{t}}{{N}_{s}}\right]$$

where *N*_*s*_ is the expression level of a given (source) node, and *N*_*t*_ is the expression level of the source node’s *t*-th target node. This computation yielded a value of approximately 5.7. In the interest of erring on the conservative side, I selected a value of 2 for this work, but believe reasonable values could easily be as high as 10.

### Estimating *ξ*

I split *ξ* into two parts (*ξ* = *αβ*), with *α* representing the proportionality between the impinging activation and the level of gene activity, and *β*, representing the proportionality between the gene’s activity and the number of transcripts that result. In order to estimate *α*, I want to know the level of activity impinging on a gene that leads to full transcriptional activation under normal (healthy) conditions. In order to do this, I considered the subset of genes that only have activating parent edges (in order to avoid the complexities associated with inhibitory interactions). For each such gene, I determined its set of transcription factors (that is, all transcription factors within the model that activate the gene), and their respective protein expression levels (taking the median expression level over all tissues and replicates in^19^, which includes paired transcriptomic and proteomic data from 32 healthy tissues taken from multiple patients). If all transcription factors were fully activated, and that activity were distributed evenly among each of their (potentially many) gene targets, then it is possible to sum up an estimate for how much activation is impinging on each gene under fully-activated conditions:

9 $${{{\Theta }}}_{i}=\sum\limits_{T\in {\{{{{\rm{TF}}}}\}}_{i}}{N}_{T}/{K}_{T}$$

where Θ_*i*_ represents the expected level of activation for gene *i* under fully activated conditions, *T* is a transcription factor belonging to the set of transcription factors that activate gene *i*, *N*_*T*_ is the protein expression level of the transcription factor, and *K*_*T*_ is the number of targets the transcription factor has. Once this is computed for each relevant gene, I simply computed an estimate of *α* as *α* = med[{Θ}]^−1^. This analysis resulted in the estimate *α* ≈ 0.25, which can be interpreted as meaning that approximately one out of four activated transcription factors goes on to activate one of its targets.

Next, I wanted to estimate *β*, the number of transcripts produced when a gene is fully activated. One simple way to estimate this is by assuming the highest transcription levels measured for each gene correspond to its full activation. We can again rely on data from^19^, in this case focusing on the transcriptomics, and extracting the 95%-ile of all TPM values for each gene (expecting that some fraction of the very highest observations are likely to be spurious outliers). These values are then used as our gene-specific *β* values.

### Estimating *γ*

In order to estimate the set of values of *γ* such that *N*_*i*_ = *γ*_*i*_*M*_*i*_ for all genes and their respective gene products, I once again turn to the paired transcriptomics and proteomics data from^19^. For each gene in each sample, the ratio of the protein expression level to its transcript expression level (*N*/*M*) was computed. Then, for each gene, the median of those ratios was extracted, yielding an estimate for each *γ* value (See Supplementary Fig. 1). In the event that no suitable *γ* value could be found this way (for example, if a gene was in the model, but not represented in^19^), the median *γ* value over all computed *γ* values was used.

### Modeling ET in breast cancer

In order to construct a reasonably comprehensive model of human signal transduction, I first downloaded all human protein-protein interactions graded B or higher from the signaling knowledgebase OmniPath^53^, along with all transcriptional interactions, and all small molecule interactions. These were compiled into a set of nodes and edges, and an LP was constructed in the manner detailed in section “SCSTA”. The python package optlang was used as an interface to the LP software package GLPK. All *N* values were taken to be proportional to their respective *M* values (taken from the TCGA BRCA data set, and mapped from Ensemble ID to the UniProt IDs used in OmniPath using UniProt’s online gene mapping tool), with *γ* values computed as described above in Methods section “Estimating γ”. In cases where a node in the model was not available in the patient’s RNAseq data, the median TPM value over all observations for that gene in ref. ^19^ was used, or failing that, the median TPM value over all observations of all genes in ref. ^19^.

OmniPath includes complexes as nodes; because proteins may be members of different complexes, and may also have signal transduction activity on their own, distributing protein copies to their respective complexes required some care. The approach taken was to begin by assuming all proteins’ expression levels (*N* values) were associated with the uncomplexed state, and then iteratively looping over every complex, discerning if there are enough uncomplexed copies of each constitutive protein to form another complex, and if so, incrementing the complex copy number by 1 while decrementing those of each constituent uncomplexed proteins. This process was repeated until no more complexes could be generated. Small molecules (which appear in some complexes) were assumed to be available in excess, and as such were ignored in this calculation.

In total, the model incorporated 14,786 genes, 11,710 proteins, 1321 complexes, 3793 small molecules, and 210,043 total interactions.

For each patient, a baseline solution was first computed. I then perturbed the network by imposing a 99% reduction in the upper bound on ER*α*-activation (that is, for the ER*α* node, I altered the constraint in Eq. (1) such that the right-hand side was 0.01 × *N*). This was meant to mimick a strong inhibitor of ER*α*. I then computed the MPS as described in section “Minimally-perturbed solution”.

It is worth noting that OmniPath allows users to restrict their downloads to only those data sets with liscences that allow for commercial use; for the purposes of this research article it is unnecessary, although in many other instances, such an option may be required. I also note that there is nothing intrinsic to the method that requires a specific network, OmniPath or otherwise. OmniPath was chosen in this work for its comprehensiveness, but other knowledgebases could have be used instead (Kegg, SIGNOR, etc.) Moreover, several methods have been developed for network inference, often relying on high-throughput experiments. Examples include the lpNet models (which use linear programming for network inference^17,18^). These and other types of unsupervised methods could be applied for network inference, and the resulting networks could be used within SCSTA studies. Minimal data requirements are outlined in Supplementary Section 1.9.

### Computing phenotype scores

SIGNOR’s phenotypes data set^20^ is comprised of a list of gene products that either up- or downregulate members of a set of phenotypes (incidentally, SIGNOR is a signaling pathway knowledgebase that forms part of OmniPath). I used it to compute a score for each phenotype for each patient using both the baseline gene expression data, and the gene expression data predicted by the MPS. Specifically, the level of activation of each gene was computed, and then transformed into protein expression values using appropriate values for *ξ* and *γ* (see Methods sections “Estimating ξ” and “Estimating γ”). In cases where the MPS resulted in gene activation levels that swung negative, the predicted copy number was set to zero (these corresponds to situations in which the sum of inhibiting edges is larger than that of activating edges, and an “overly-inhibited” gene is still presumed to produce no transcripts or proteins). Then, weighted sums associated with each phenotype were computed; if a given gene product upregulates a given phenotype, the corresponding protein expression value was added, and when one downregulates a phenotype, it was subtracted.

Many of these phenotypes correspond to closely related behaviors, and as such were further grouped. For example, the phenotypes “Metabolism,” “Glycolysis,” and “Oxidative phosphorylation” are all clearly associated with metabolism, and more generally, are associated with enhanced cell proliferation. Not only can they be grouped together, they can also reasonably be grouped with the phenotypes “Proliferation” and “Cell growth,” among others. Accordingly, a second round of weighted sums, corresponding to just six behaviors (proliferation, cell death, cell cycle, immune, stemness, and metastasis) were constructed. These are detailed in Supplementary Table 9.

Finally, because the absolute values of each score can vary wildly (in part because they are composed of different numbers of phenotypes, and those are composed of different numbers of gene products) the mean and standard deviation of the computed baseline scores for each behavior were found, and used to standardize both the baseline scores and the MPS scores such that:

10 $${X}^{* }=\left(X-{\mu }_{{X}_{{{{\rm{baseline}}}}}}\right)/{\sigma }_{{X}_{{{{\rm{baseline}}}}}}$$

where *X* represents some raw score, and X^*^ its corresponding standardized score, ${\mu }_{{X}_{{{{\rm{baseline}}}}}}$ represents the mean of the raw baseline scores, and ${\sigma }_{{X}_{{{{\rm{baseline}}}}}}$ represents the standard deviation of the raw baseline scores. This essentially z-scores the baseline scores, and transforms the MPS scores in terms of standard variates relative to the baseline scores.

### Imputation of feature values for TCGA patients

Several clinical and pathological features of interest were missing for many of the TCGA patients. These included immunohistochemistry (IHC) statuses for estrogen receptor (ER), progesterone receptor (PR), and Her2/neu, as well as T, N, and M stages.

The IHC statuses, when unavailable or equivocal, were imputed based on available RNAseq data. For each of the genes ESR1, PGR, and HER2, all patients with positive or negative statuses were identified and their corresponding gene expression levels (unstranded TPM) were extracted. Cutoffs were then selected for each gene in order to maximize the Youden’s J-statistic. This was done by iteratively looping over each gene TPM for each patient in the set, using it as a cutoff to predict IHC status, then computing the J-statistic based on the known IHC statuses, and ultimately selecting the cutoff with the highest J-statistic overall. The corresponding cutoffs were 2029 for ESR1, 390 for PGR, and 24311 for Her2. These were then used to impute any missing or equivocal IHC statuses.

Missing T, N, and M stages (including indeterminate values such as those labeled TX, NX, or MX) were imputed based on the mode of the available data. Substages were ignored (e.g., T2a, T3b, *etc*. were treated as T2, T3, etc.), and then the most frequent stage of those available was computed and used to impute any missing values for the remaining patients.

### Survival analysis

The Python package lifelines was used to perform Cox regressions and Kaplan–Meier fits. Overall survival data was extracted from TCGA, truncated at 10 years, and used in each analysis.

A Cox proportional hazards model was used to investigate whether the changes in each behavior score were associated with risk of death. I include as covariates the change in each behavior score (binarized at the median value of each score change), progesterone receptor (PR) and HER2 statuses, T-, N-, and M-stages, age at diagnosis (z-scored), and *ESR1* expression level (wherein the TPM value was extracted, one was added to it, and the result was logged and z-scored). The later was included in order to ensure that any risk associated with the predicted changes in behavior was not just a trivial association with high or low ER*α* expression, since the model itself is predicated (in part) on ER*α* expression.

Because the change in proliferation score was found to be associated with risk of death, a univariate Cox model was also performed, and an associated Kaplan–Meier plot was produced (see Fig. 3b).

Additional Cox regressions were performed to investigate the impact of including the pretreatment proliferation score. These included a bivariate analysis (pretreatment proliferation score, and the change in score, both binarized at their respective medians), and a more comprehensive model that also included age, T-, N-, and M-stages, PR and HER2 statuses, and *ESR1* expression. Finally, patients were grouped into four categories, “+/+,” “+/−,” “−/+,” and “−/−” based on their binarized change in proliferation score and binarized pretreatment proliferation score, respectively, and a Kaplan–meier plot was produced (see Supplementary Fig. 6).

### Determining drivers of changes in behavior scores

In order to determine which gene-associated edges are most responsible for the predicted population-level changes in each behavior score, I first computed quantity *π*, representing the per-patient score change in response to a change in activity of a given edge:

11 $${\pi }_{i,k}^{l}={\gamma }_{i}{\xi }_{i}\left(\sum\limits_{j}{f}_{i}^{l}{\delta }_{i}^{l}{p}_{i,j}{b}_{j,k}\right)$$

where *l* represents the index of a given patient, *γ*_*i*_ is the gene product to transcript ratio for the gene with parent edge *i*, *ξ*_*i*_ is its activation to transcript ratio, ${\delta }_{i}^{l}={e}_{i}^{{{{\rm{MPS}}}}}-{e}_{i}^{{{{\rm{baseline}}}}}$ is the change in activation of edge *i* in patient *l*, *p*_*i*,*j*_ is the weighting of the gene product associated with edge *i* in phenotype *j* (either 1 if it upregulates the phenotype, − 1 if it down-regulates, or 0 if it does not affect the phenotype), and *b*_*j*,*k*_ is the weighting of phenotype *j* on behavior score *k* (analogous to *p*_*i*,*j*_, *b*_*j*,*k*_ takes values 1, − 1, or 0; see Supplementary Table 9). The quantity ${f}_{i}^{l}$ corrects for gene activations that swing negative in the MPS (see Methods section “Computing phenotype scores”); it is the fraction of the change in the activity of the gene associated with edge *i* that is non-negative. I then used these values to compute the fraction of the total absolute score change (over all genes and all patients) that was associated with each edge:

12 $${\phi }_{i,k}=\frac{{\sum }_{{{{\rm{patient}}}}\,l}| | {\pi }_{i,k}^{l}| | }{{\sum }_{{{{\rm{patient}}}}\,l}{\sum }_{{{{\rm{edge}}}}\,i}| | {\pi }_{i,k}^{l}| | }$$

Higher values of *ϕ*_*i*,*k*_ indicate that edge *i* is responsible for a larger fraction of the total change in score *k*.

### Maximally-dysregulated pathways

Given a set of paired baseline and MPS solutions, the MDP is the pathway that connects some edge in the network to a perturbed node such that each edge along the pathway best explains the change in the following downstream edge. In order to compute this, I first introduce the quantity *ψ*_*i*,*n*_, representing the fraction of the sum over all patients of the absolute values of the changes in activation flowing into or out of each node, *n*, associated with each child or parent edge *i*:

13 $${\psi }_{i,n}=\frac{{\sum }_{{{{\rm{patient}}}}\,l}{\delta }_{i,n}^{l}}{{\sum }_{{{{\rm{patient}}}}\,l}{\sum }_{{{{\rm{edge}}}}\,i}| | {\delta }_{i,n}^{l}| | }$$

where ${\delta }_{i,n}^{l}={e}_{i}^{{{{\rm{MPS}}}}}-{e}_{i}^{{{{\rm{baseline}}}}}$ is the change in edge *i* (associated with node *n*) under the perturbation. These *ψ* values can be used to construct the MDP straightforwardly.

First, an edge (denoted *e*_1_) is specified. This may be, for example, the transcriptional regulation of the some gene by some transcription factor. I then compute the *ψ* values of all incoming and outgoing edges connecting to *e*_1_’s parent node (the transcription factor, denoted *n*_1_). From there, I determine which other edges connected to *n*_1_ could possibly give rise to the observed change in *e*_1_. For example if *e*_1_ tends to decrease (negative *ψ*), then I am only interested in other edges connected to *n*_1_ that can explain that decrease, which include child edges that increase in activity (positive *ψ* values, representing a diversion of activity to some other pathway), an activating parent edge that decreases in activity, or an inhibiting parent edge that increases in activity. Of those possibilities, the one with the largest absolute value for *ψ* is selected, and labeled as *e*_2_, with its parent being labeled as *n*_2_. This process is repeated iteratively (explicitly excluding the possibility of traversing the same edge twice) until the perturbed node (e.g., ER*α*) is reached.

### Modeling ET in 25 patients with pre- and post-treatment transcriptomes

Transcriptomis data associated with ref. ^48^ was downloaded from ArrayExpress^54^ (accession: E-MTAB-9917). Patients for which at least one sample was associated with a pre-treatment timepoint were selected. Log_2_ normalized counts were then transformed to TPM, and SCSTA was used to model ET as described above in Methods section “Modeling ET in Breast Cancer”.

### Linear Regression to predict changes in gene expression under ET

As a comparator for our SCSTA calculations, a multiple linear regression model was developed to predict post-ET gene expression from pre-ET expression. In each case, the earliest post-treatment sample was selected. The LinearRegression implementation in python’s sklearn package was used. The model was trained to predict an entire post-treatment transcriptome from a pretreatment transcriptome. A leave-one-out approach was used, wherein patient *i*’s gene expression was predicted using their pre-treatment expression data, and a model trained on all *j* ≠ *i* patients’ pre- and post-treatment data (the training set). I tried using TMP values directly as inputs and outputs, as well as log_2_-transformed values, and found the model performed better with logged values, which was ultimately used as the comparator described in section “Direct comparison of SCSTA predictions with measured post-ET data”.

### Supplementary information

Supplemental Information

## Data Availability

This study utilized only previously-published and freely available data sets (https://portal.gdc.cancer.gov/projects/TCGA-BRCA, and tables published with^19^); no new primary data (*e.g* transcriptomics, proteomics, *etc*.) were generated.

## Code availability

The underlying code for this study is not publicly available but may be made available to qualified researchers on reasonable request from SimBioSys Inc.
