# Supplementary material for: Self-consistent signal transduction analysis for modeling context-specific signaling cascades and perturbations
Source: NPJ Syst Biol Appl. 2024 Jul 19;10:78. doi: 10.1038/s41540-024-00404-x (PMC11271576; doi:10.1038/s41540-024-00404-x)
Supplement: Supplementary file 1 — Supplemental Information [file 41540_2024_404_MOESM1_ESM.pdf]

Self-consistent Signal Transduction Analysis for Modeling Context-Specific Signaling Cascades and Perturbations Supplemental Information

John Cole[1]

[1]SimBioSys Inc., 60 Hazelwood Dr., Suite 230D, Champaign IL, 61821

July 9, 2024

1 Supporting Information

1.1 Details on various assumptions and formalisms

The work presented here rests on several assumptions and/or formalisms. The overarching goal is to develop a method that is capable of rapid simulation of the signaling pathways that are active within a given biological sample, regardless of tissue type. As such, the method must be comprehensive (containing all or most known pathways), and it must be "parameter-free," or nearly so, so that it does not require training sets to tune it for individual tissue types, regulatory states, or genotypes. As noted in the introduction, constraint-based models, like those long used to study metabolic networks, offer both, but they do come with tradeoffs, including a steady-state approximation.

The key mathematical formalism used here revolves around linear programming. LPs have been widely-used, primarily in operations research, before being leveraged to study biological networks. The core idea is that one wants to optimize (either maximize or minimize) some linear combination of variables, subject to constraints on those variables. In the present work, those constraints include: 1) the sum of activities impinging on each gene must be linearly related to the gene's expression level; and 2) the activation level of each protein must not exceed the expression level of the protein (which can be interpreted to mean that there can not be more activated proteins than there are actual proteins). The main linear combination that is minimized is the total sum of activations (in the "baseline" solution, see Section 2.1.1 in the main text), or the absolute deviation from the baseline solution (in the minimally-perturbed solution described in Section 2.1.2 in the main text).

Of course, several assumptions are made along the way. The simplest of these include: 1) protein and transcript expression levels are linearly related (see Section 4.2 in the main text); 2) gene activation and transcript expression

are linearly related (see Section 4.1); and 3) leveraging transcriptomics and proteomics data from healthy tissues is a reasonable way to estimate those linear relationships, even among diseased samples. More subtle assumptions that are made include: 4) signaling activity is approximately conserved (meaning activation gets "passed on" from molecule to molecule through the network); 5) constitutive activation and spontaneous deactivation of signaling proteins and genes is biologically disfavored (Section 2.1.1 in the main text); 6) signaling networks tend to be efficient (that is, they tend toward a state of minimal total activity, see Section 2.1.1 in the main text and Supplementary Section 1.2); and 7) signaling networks are minimally impacted by perturbations (Section 2.1.2 in the main text).

1.2 Biases relating to parsimonious solutions in SCSTA

As noted in Section 2.1.1 of the main manuscript, the parsimony objective introduces certain biases in the solutions generated by SCSTA. In particular, it creates a tendency toward increased constitutive activation at or near the gene nodes. To see why, consider the trivial network motif shown in Supplementary Figure 8A. If we assume that the impinging activation of gene G is required to be 1, we immediately see that the minimal total activity flowing that can flow through this network (the parsimonious solution) occurs when $e_{CA,G} = 1$ and all other edges take the value 0. Barring that, we also see that the next-most parsimonious solution occurs when $e_{CA,C} = 1$, $e_{C\to G} = 1$, and all other edges are zero. Indeed, we can continue up the pathway like this, finding increasingly-less parsimonious solutions. This observation led to the introduction of the introduction of objective c_1.

This is not the only unintentional bias introduced by the parsimonious objective, however. Another important one is a general tendency toward decreased reliance on inhibitory interactions. Consider the network motif in Supplementary Figure 8B, and again assume we require the total activation impinging on G to be 1. In this case, barring constitutive activations, the parsimonious solution will simply involve activity flowing through the left-hand pathway (*e.g.* $e_{A\to B} = e_{B\to G} = 1$), with the right-hand pathway being unused. Indeed, for the right-hand pathway to be active requires higher levels of activity in the left-hand pathway, and larger total pathway activity in general. The bias against such pathway activations manifests itself in a particularly striking way within the data presented in the main manuscript. For a gene to be predicted to be upregulated under inhibition of some upstream protein, the connecting pathway must involve at least one inhibitory interaction. As a result, the parsimonious objective's tendency to minimize use of such pathways leads to a decreased tendency to predict upregulation of genes, as seen in Figure 5 in the main manuscript.

In light of the overall performance of the model, and its apparent ability to prognosticate better and worse patient outcomes, the parsimonious objective used in this work still represents a reasonable starting point. Nevertheless, I believe that other objectives are worth investigating in future work. One might expect, for example, that there should exist evolutionary pressure against expression of signaling proteins that are not active, and that in turn most expressed proteins should be active at or near their maximal capacity; this might lead one to consider an objective in which total activity was maximized, perhaps using some weighting based on the proteins' degree of upregulation

relative to healthy tissues.

1.3 Full self-consistency of the MPS

Here I suggest to the reader a few possible methods for ensuring full self-consistency in an MPS.

The simplest way to ensure full self-consistency may be an iterative approach, wherein an MPS solutions is sought, new N values are computed, and then they are used to update the relevant constraints, leading to yet another MPS solution (still being being minimally perturbed from the original baseline solution). This can be repeated until the changes in M values fall below some tolerance, and the solution is considered to be converged.

A second approach would be to reformulate the model such that the N values themselves are implicitly defined in terms of the M values. For example the constraint in Equation 1 of the main manuscript could be rewritten such that the right-hand side is less than or equal to the product of γ and the corresponding node's transcriptional activation (that is, the left hand side of Equation 3 in the main manuscript):

$$\sum_{e_p^n} w_p^n e_p^n \leq \gamma\xi\Big(- e_{\text{SD}} + \sum_{e_p^g} w_p^g e_p^g \Big) \tag{1}$$

where e_p^n represents the parent edges of node n, and e_p^g represents the parents edges of the corresponding gene. This is problematic because it does not, in and of itself, account for the distribution of gene products to complexes. Nevertheless, such an approach can be saved by also making this distribution implicit. For example, if gene product A performs interactions on its own, and is also a subunit of complexes B, C, *etc.*, and they engage in other interactions, then if we require:

$$\sum_{n\in\{A,B,C,\ldots\}} \sum_{e_p^n} w_p^n e_p^n \leq \gamma\xi\Big(- e_{g,\text{SD}} + \sum_{e_p^g} w_p^g e_p^g \Big) \tag{2}$$

and do the same for all other proteins in every complex, we effectively limit the amount of activation into each gene product and all of its associated complexes such that the total activation is bound by the expression level of the respective gene products.

1.4 Pretreatment behaviors tend to align with expected trends among ER-positive and ER-negative patients

ER-positive patients have better prognoses than ER-negative patients. They tend to be less malignant and of lower grade, less proliferative, less apoptotic [1], show better differentiation (less stemmness) [2, 3], and show less immune infiltrate [4]. I investigated whether the TCGA patients showed the same types of trends prior to simulated ET. Patients were categorized as either ER-positive or -negative based on their reported immunohistochemistry (IHC) status, or, when unavailable or equivocal, based on gene expression data (see Methods Section 4.5 in the

Main Manuscript). Welch't t-tests were then performed to determine whether each baseline behavior score differed between the two groups (see Supplementary Table 1). Significant differences were found in the cell death, cell cycle, immune, and stemness scores, each of which, except for the immune score, followed the expected trend (I note that the "Immune response," phenotype, and several others that comprise the immune score, showed the expected trend. An insignificant difference was observed in the proliferation score, but it did follow the expected trend. Surprisingly, the metastasis score showed a significant difference, but it was in the opposite direction of that which was expected.

The immune score used in this work is comprised of 20 SIGNOR phenotypes, describing a range of different behaviors critical to immune system function. Prior to simulated ET, the ER-positive patients tended to have significantly larger immune scores than the ER-negative patients, in stark contrast to the general understanding within the field. As such, a more detailed analysis was carried out in which the values of each of SIGNOR's immune-related phenotypes were computed and t-tests were performed to determine which phenotypes showed significant differences. Broadly speaking, the ER-positive patients tended to show: lower "Macrophage activation", lower "M1 polarization" and higher "M2 polarization", and higher "Macrophage differentiation"; lower "T-reg differentiation"; higher "T-lymphocyte differentiation" and "T-cell activation"; higher "B-lymphocyte differentiation" but lower "B-cell maturation"; higher "Basophil differentiation", and as noted above, lower "Immune response" (see Supplementary Table 2).

The metastasis score is much more problematic, and indeed I consider it essentially meaningless. It is composed of only a single SIGNOR phenotype, and not only does it not not follow the expected pretreatment trend, even the post-treatment score fails to align with reported M-stage (see Supplementary Figure 2). It is possible that the inclusion of multiple phenotypes in the other scores increases their robustness.

1.5 Epithelian-mesenchymal transition portends low sensitivity to endocrine therapy

Epithelial-mesenchymal transition is known to confer insensitivity to endocrine therapy. I found that among ER-positive patients, when ERα is inhibited, the stemness score, which includes genes associated with EMT, tends to increase. As shown in Supplementary Section 1.7 and Supplementary Table 7, the predominant mode through which this occurs is via decreased E-cadherin expression, itself a negative regulator of EMT. This means that as ER-signaling decreases, EMT is predicted to increase. Conversely, I considered the ER-positive patients with high vs. low pre-treatment EMT scores. I found that the mean change in proliferation under ET among patients with high pre-treatment EMT scores (that is, those with EMT scores greater than the median value over all patients) was -1.15 standard variates, while those with low EMT scores was -1.53 standard variates. A Welch's t-test gives a p-value of 0.0017, indicating that patients with high pre-treatment EMT scores tended to be significantly less responsive, in terms of proliferation, to ET than those with low scores.

In [5], it was shown that the expression of genes associated with several phenotypic traits were altered in an MCF-7-derived cell line with silenced estrogen receptor. In order to directly test the model's capacity to recapitulate

these findings, I downloaded a dataset of RNAseq data for nearly 700 cell lines [6, 7], and simulated ERα inhibition using the MCF-7. The model tended to predict relatively small numbers of differentially-regulated genes, and predicted only a single gene to be upregulated (see Supplementary Section 1.2 above). Although not specifically noted in [5], this upregulated gene was *CXCL8*, a known enhancer of EMT [8]. Of the genes that were observed to be downregulated in [5], the model correctly identified *ERBB4* (associated with endocrine resistance), *PRLR* (luminal and epithelial phenotypes), *TFF1* (luminal and invasion/metastasis phenotypes), *HOXA1* and *HOXC6* (invasion/metastasis), *KRT15* and *KRT19* (epithelial phenotype), and *CLDN4* (epithelial phenotype). For each of these genes, the model predicted complete loss of expression. Surprisingly, E-cadherin expression was predicted to be unchanged, despite it being the primary driver of the stemness score change among TCGA patients. While imperfect, these combined results point toward the loss of luminal and epithelial phenotypes, increased invasiveness and metastatic potential, and increased endocrine resistance in estrogen receptor silenced MCF-7 cells.

I next sought RNAseq data associated with ER-positive breast cancer cell lines with high expression levels of Snail, Slug, and Twist—all markers of EMT [9]. I again turned to data from [6, 7], and subsetted it to only the breast cancer lines. I then computed the median TPM for ESR1 expression, finding a value of 2. I next consulted the literature to determine which ER-positive cell lines are in common use. I found that MCF-7, T47D, and ZR-75-1 are the most common [10], and of them, the lowest ESR1 expression level was that of ZR-75-1, taking a value of 4 TPM in the transcriptomics dataset—still larger than the median value. I took this value of 4 TPM to be my cutoff for ESR1 positivity, and then sought the cell lines with the largest expression levels of Snail, Slug, and Twist. The highest Slug and Twist expression levels occurred in the cell line JIMT-1 (with a Slug expression of 130 TPM, Twist expression of 35 TPM, and ESR1 expression level of 7 TPM), while the highest Snail expression level occurred in BT-483 (with Snail expression of 20, and ESR1 expression of 29). I then simulated the impact of ET in each cell line, finding that neither cell line's proliferation score changed appreciably. JIMT-1's proliferation score decreased by about 8.1×10^{-3}%, while BT-483's proliferation score decreased by about 0.6%. I note that JIMT-1 is not generally considered ER-positive, despite its ESR1 expression level being higher than that of ZR-75-1 in this dataset. While by no means conclusive, this indicates that (arguably) ER-positive cell lines with high expression levels of EMT markers should indeed be insensitive to ET.

1.6 Correlations among covariates used in survival analysis

In order to determine which, if any, covariates in the survival analysis were correlated, I computed the Spearman correlation coefficient between each covariate pair. Only one particularly strong correlation (*i.e.* having a correlation coefficient, ρ_S, with magnitude greater than 0.5) was observed. This was observed between the predicted change in stemness score and *ESR1* expression level, with a correlation coefficient of 0.61. This indicates that tumors with high *ESR1* over-expression are predicted to show larger increases in stem-like behaviors during ET. Beyond that, other correlations tended to be small ($|\rho_S| \leq 0.2$ in magnitude) or modest ($0.2 < |\rho_S| \leq 0.5$).

As noted in Section 2.2.1 of the main manuscript, the proliferation score change showed only modest Spearman correlation coefficients with *ESR1* expression, and the change in stemness score. The change in cell death score followed a similar trend, again showing only Spearman correlations between it and both *ESR1* expression and the change in stemness score (ρ_S values of 0.26 and 0.34, respectively). The change in cell cycling behaviors showed only small spearman correlations. The change in immune score, like proliferation and death, was modestly correlated with the *ESR1* expression and the change in stemness (ρ_S values of 0.23 and 0.25, respectively). In addition to those noted above, the change in stemness score was also modestly correlated with patient age at diagnosis ($\rho_S = 0.27$). The change in metastasis score only showed a single modest correlation with *ESR1* expression ($\rho_S = 0.26$).

Finally, among the clinical data covariates (T-, N-, and M-stages, ER, PR, and Her2 statuses, and age at diagnosis), modest correlations were observed between T-stage and N-stage ($\rho_S = 0.28$), and between age at diagnosis and *ESR1* expression.

1.7 Pathway Analysis of other behaviors

Cell Death. Computing the ϕ values for the cell death behavior yielded a diverse mixture of both pro- and anti-cell death activations. The three largest ϕ values that tend to lead to increased cell death are closely related to those that also led to decreased proliferation described in the main manuscript, namely the decreased activations of the genes *AKT1*, *AKT2*, and *HK1* (with ϕ values of approximately 0.12, 0.13, and 0.077, respectively). The three largest ϕ values that led to decreased cell death included decreases in the transcriptional activations of: *FOXA1* by FOS ($\phi \approx 0.087$); *HIP1* by TFAP2A ($\phi \approx 0.082$); and *CASP3* by TP53 ($\phi \approx 0.057$).

The MDP leading to the decrease in *FOXA1*'s transcriptional activation by FOS involved only one additional player, JUN (see Supplementary Figure 7). *FOXA1* acts as an activator of both SIGNOR's "Apoptosis" and "Cell death" phenotypes. Under ET, the model predicts that the loss of ERα's activation of JUN is partially offset by the redirection of FOS's activation away from *FOXA1* transcriptional activation, leading to a decrease in *FOXA1* expression, and in turn a decrease in our cell death score.

The MDP leading to the decrease in *HIP1*'s transcriptional activation by TFAP2A involved two additional steps (see Supplementary Figure 7). Like *FOXA1*, *HIP1* acts as an activator of both SIGNOR's "Apoptosis" and "Cell death" phenotypes. Under ET, the model predicts a decrease in ERα's activation of GNA13, which is partially offset by increased activation of GNA13 by F2R (the first two steps in the pathway leading to decreased *HK1* expression). Then, in addition to the aforementioned redirection of F2R's activity away from EGFR (Figure 4 in the main manuscript), this increased F2R activity is also partially supported by redirection of TFAP2A's activity away from transcriptional activation of *HIP1*. This leads to decreased *HIP1* expression, and a decrease in the cell death score.

Finally, the MDP leading to the decrease in *CASP3*'s transcriptional activation by TP53 involves one additional player, COL18A1 (see Supplementary Figure 7). *CASP3*, like *FOXA1* and *HIP1*, acts as an activator of both SIGNOR's "Apoptosis" and "Cell death" phenotypes, among several others. Under ET, the model predicts a

decrease in ERα's activation of COL18A1, which is partially offset by redirection of TP53's activity away from *CASP3* transcriptional activation.

Leveraging the data from [11], *FOXA1*, *HIP1*, and *CASP3* all show evidence of downregulation under ET, although only *FOXA1* is significantly so (with 1-sided t-test p-values of 0.0011, 0.15 and 0.19, respectively).

Cell Cycle. The ϕ-values for the cell cyling score indicated that approximately 75% of the observed score change under ET can be attributed to just three transcriptional activations. Each is associated with the direct activation of a target gene by ERα, although their impact on the score varies. The most important ($\phi \approx 0.34$) corresponds to the transcriptional activation of *E2F1*, which is an activator of SIGNOR's "G1/S transition" phenotype, and in turn, an activator of our cell cycle score. The second most important ($\phi \approx 0.26$) corresponds to the transcriptional activation of *CDKN1A*, which is included as an inhibitor of SIGNOR's "Cell cycle progress" phenotype, as well as an activator of its "Cell cycle block" and "Cell cycle exit" phenotypes, and thus a strong inhibitor of our cell cycle score. The third most important ($\phi \approx 0.15$) corresponds to the transcriptional activation of *CCND3*, which is included as an activator of SIGNOR's "Cell cycle exit" phenotype, and thus an inhibitor of our cell cycle score. All told, the loss of ERα activity under ET is predicted to lead to a decrease in expression of each of these genes, giving rise to a mixed effect wherein the decrease in *E2F1* decreases cell cycle activity, while the decreases in *CDKN1A* and *CCND3* lead to increases.

As before, I leveraged the data from [11], and found that *E2F1*, *CDKN1A*, and *CCND3* all show evidence of downregulation under ET, but that only *E2F1* is significantly downregulated (with 1-sided t-test p-values of 6.4×10^{-5}, 0.30 and 0.31, respectively).

Immune. The observed change in the immune activity score under ET was almost solely associated with a predicted decrease in transcriptional repression of *IL-8* by ERα ($\phi \approx 0.94$). *IL-8* is an activator of SIGNOR's "Inflammation" phenotype, which is, in turn, an activator of the immune activity score. *IL-8* expression is known to negatively correlate with ERα expression, be regulated by both ERα and FOXA1 among others, and has been associated with increased invasiveness, proliferation, metastatic potential, and endocrine resistance [12–14]. Nevertheless, no additional evidence could be found to directly support the predicted increase in *IL-8* expression or associated inflammation within the breast TME under ET, and in fact, the data in [11] indicates an (insignificant) decrease in *IL-8* expression ($p \approx 0.23$).

Stemness. The vast majority of the change in stemness score under ET was associated with two transcriptional activations, namely those of *CDH1* by ERα ($\phi \approx 0.52$), and *NANOG* by a complex comprised of RARA and RXRA ($\phi \approx 0.42$). *CDH1* is an inhibitor of SIGNOR's "Epithelial-mesenchymal transition" phenotype, and in turn an inhibitor of our stemness score, while *NANOG* is an activator of SIGNOR's "Pluripotency" phenotype, and thus an activator of our score.

The loss of ERα activity under ET is predicted to decrease *CHD1* expression, leading to an increase in stemness. The predicted regulation of *NANOG* is more subtle (see Supplementary Figure 7). The MDS that proceeds from *NANOG* involves: the loss of activation of FOX1A by ERα; the subsequent loss of activation of FOX1A's downstream target Vitronectin; reallocation of the activity of Thrombospondin-1 away from its inhibition of the RARA:RXRA complex toward activation of Vitronectin; ultimately leading to enhanced transcriptional activation of *NANOG* and the predicted increase in the stemness score.

I again leveraged the data from [11], and found that the gene *CDH1* was significantly downregulation under ET ($p \approx 0.016$), and that, although not significant, *NANOG* showed a trend toward upregulation (a one-sided t-test for upregulation yielded a p-value of 0.25).

Metastasis. Despite deep reservations about the value of the metastatic potential score (see Supplementary Section 1.4 above), I nevertheless analyzed the drivers of its predicted change under ET. The dominant cause for the predicted change in the metastatic potential score was a predicted decrease in transcriptional inhibition of *MET* by FOX1A ($\phi \approx 0.89$; see Supplementary Figure 7). *MET* is an activator of SIGNOR's "Metastasis" phenotype, and is thus an activator of our metastatic potential score; under ET, the model predicts that activation of FOX1A by ERα will decrease, and in turn, inhibition of the *MET* gene will also decrease, leading to enhanced transcription, and increased metastatic potential. The data from [11] indeed shows significant upregulation of *MET* under ET ($p \approx 0.0097$).

I note that *MET* has been associated with basal-like breast cancers, and has been considered as a therapeutic target in that context [15]. It was also noted in Section 2.2 of the main manuscript that the increase in the metastasis and immune scores may be related to ET causing a shift toward a more basal-like state. It is possible that part of this shift may be through enhanced *MET* expression. This, of course, raises the possibility that some ER-positive patients may benefit from dual-blockade of ERα and *MET*.

1.8 Proposed microenvironmental SCSTA

The bulk RNAseq samples used in this manuscript represent mixtures of various cell types. In its current form, the model can not account for the types of intercellular signaling that may be present (*e.g.* immune evasion via PD-1/PD-L1, *etc.*). Nevertheless, one exciting avenue presents itself. One could imagine employing a strategy in which the bulk RNAseq is first deconvolved and purified in order to reveal cell-type specific mixing fractions and associated expression profiles. Methods like CIBERSORTx, among others [16], could be brought to bear in this regard. Then, a compartmentalized SCSTA model could be constructed, such that each cell type represents a different compartment within a shared microenvironment (see Supplementary Figure 10). Each compartment could be thought of as its own sub-model, with its own gene expression-based sets of constraints. Importantly, however, the compartments can be coupled through signaling interactions among secreted and/or cell surface proteins. Specifically, intracellular signaling proteins within each cell type would be capable of interacting with other proteins of the same cell type, but the secreted or surface-bound proteins would be able to engage in interactions with the surface-bound proteins

of any cell-type in the full model. Solution of this compartmentalized model would proceed in much the same way as outlined in this manuscript. This would enable the model to predict the effects of inter-cellular signals, and simultaneously serve to mitigate errors or uncertainty that may otherwise arise (e.g. if the model predicts pathways activity through proteins known to be expressed by some cell types present in the bulk RNA sample, but not in the tissue of interest).

1.9 Minimal Data Requirements for SCSTA

Several minimal data requirements exist for the use of the methods presented here. SCSTA requires some sort of directed graph representing the signal transduction network; as noted in the main manuscript, this could come from either an existing knowledgebase (like OmniPath or some similar repository), or be constructed through some sort of network reconstruction algorithm. SCSTA, as envisioned here, also requires gene expression data, including at a minimum, RNAseq data, although paired transcriptomics and proteomics could be leveraged. If proteomics is not available, some sort of mapping between transcript expression and protein expression is required; here I used estimates for protein-to-transcript ratios extracted from [17]. Semi-quantitative transcriptomics or proteomics data, such as microarray data, or multiplexed immunofluorescence would require some sort of data normalization scheme in order to map measured values directly to expression level. Finally, I note that significant variability exists in gene expression and in patient outcomes; in order to make meaningful claims connecting predicted pathway activities or perturbations to patient survival, researchers will, in general, require relatively large numbers of patients' transcriptomes.

1.10 Supplementary Figures

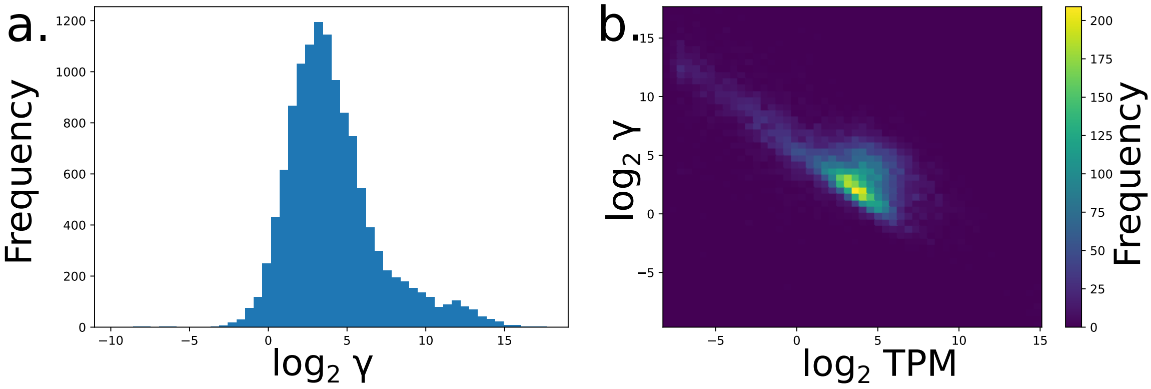

Supplementary Figure 1: **Histograms representing γ parameters extracted using paired healthy tissue proteomics and transcriptomics data from [17].** Panel a. shows the distribution of $\log_2 \gamma$ values, while panel b. shows the bivariate distribution of $\log_2 \gamma$ values and corresponding median gene expression levels (in $\log_2$ TPM) measured among the same healthy tissues from [17]. The apparent inverse relationship is due to the fact that the γ values themselves are defined as a ratio of observed protein expression levels to observed transcript levels.

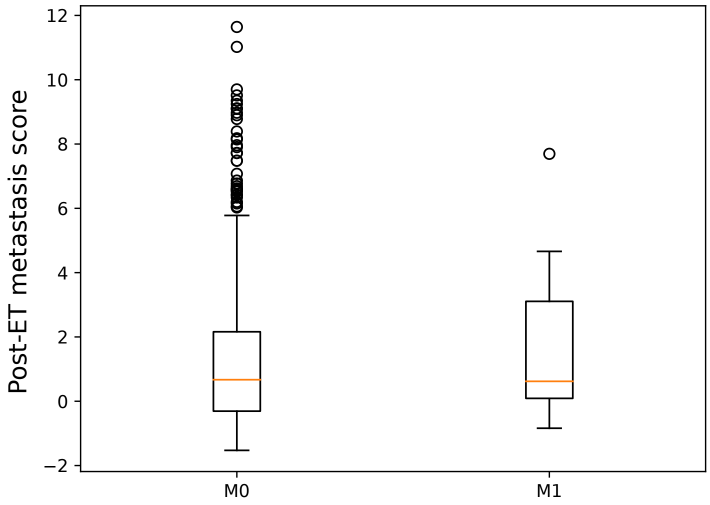

Supplementary Figure 2: **Boxplots of post-ET metastatic potential scores, stratified by M-stage.** No clear difference is evident. A Welch's t-test was performed, resulting in a p-value of 0.60.

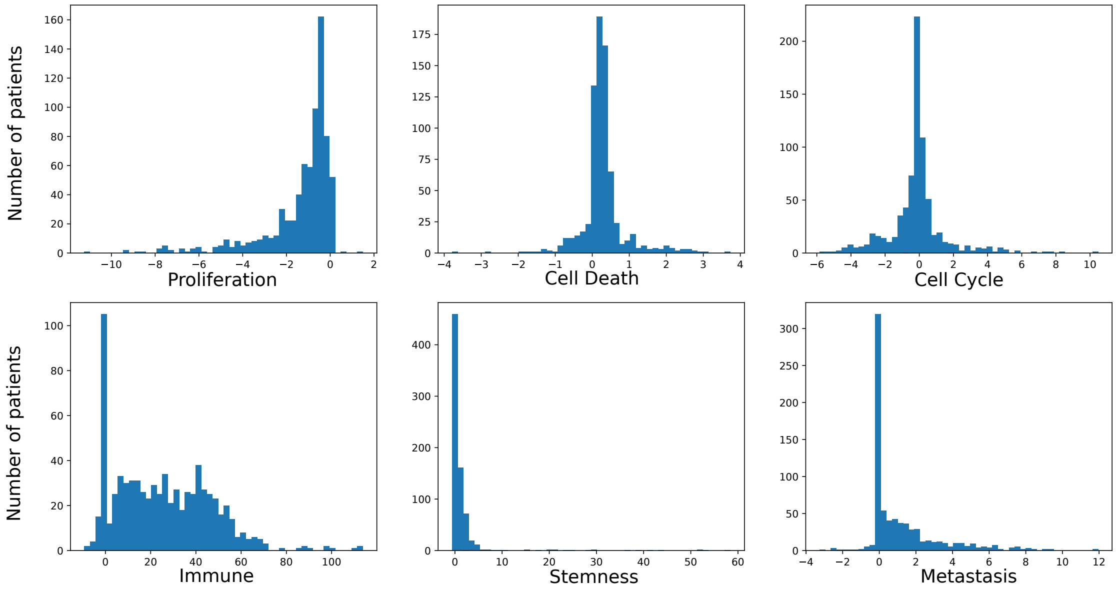

Supplementary Figure 3: **Histograms of predicted changes in behavior scores.** For each of the six behavior scores, the predicted changes (post-ET minus pre-ET) among ER-positive patients is shown.

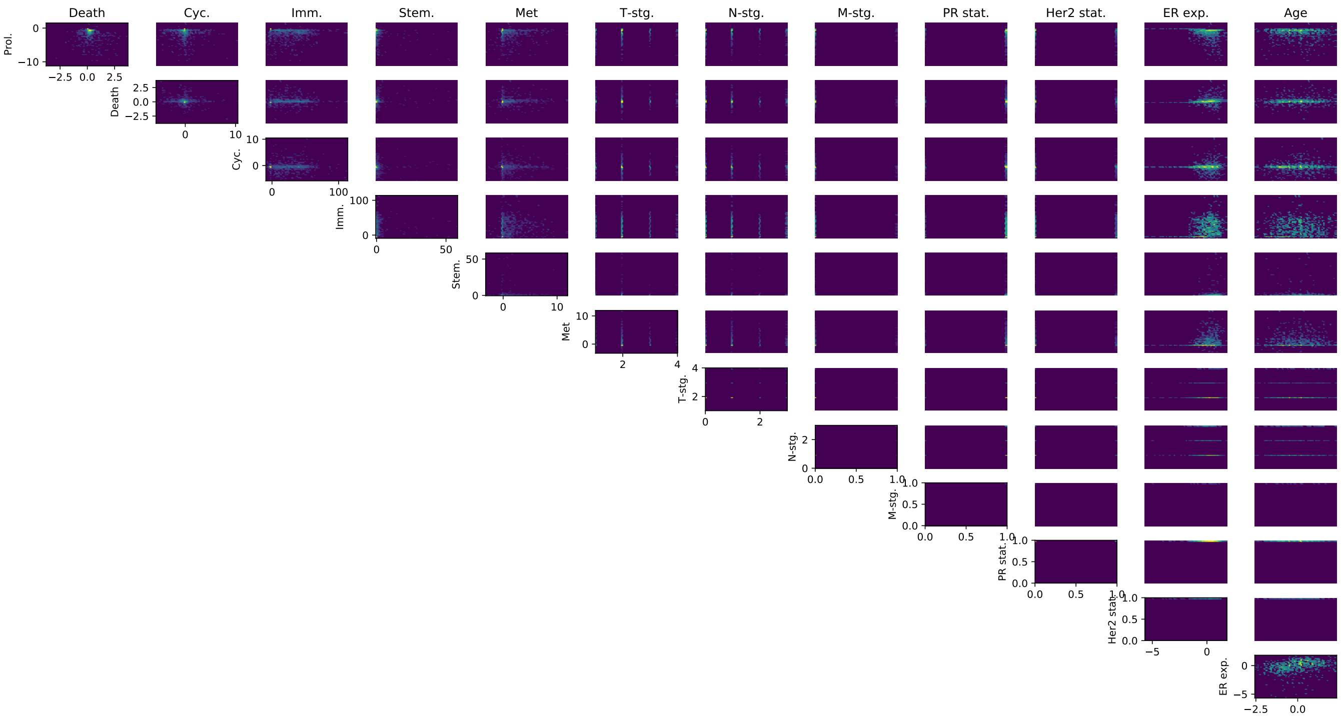

Supplementary Figure 4: **2D histograms showing the bivariate distributions of each pair of predicted score changes and clinical features used in Section 2.2.1 of the main manuscript.** In the vast majority of covariate pairs, no obvious trend is observed. In certain select pairs, modest correlations are found, and in one pair—namely the change in stemness score and *ESR1* expression level—a large correlation is observed (see Supplementary Section 1.6).

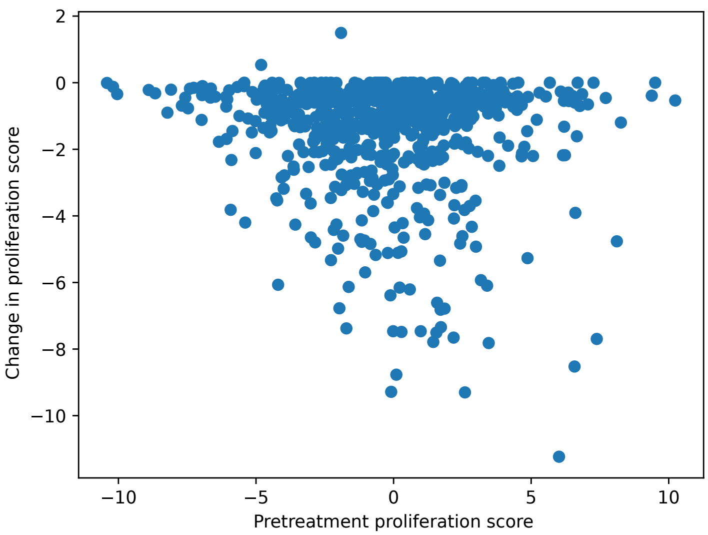

Supplementary Figure 5: **Scatter plot of predicted change in proliferation score** *vs.* **pre-treatment score.** Each point represents an ER-positive patient with x-value indicating the pre-treatment score, and y-value indicating the predicted change in score.

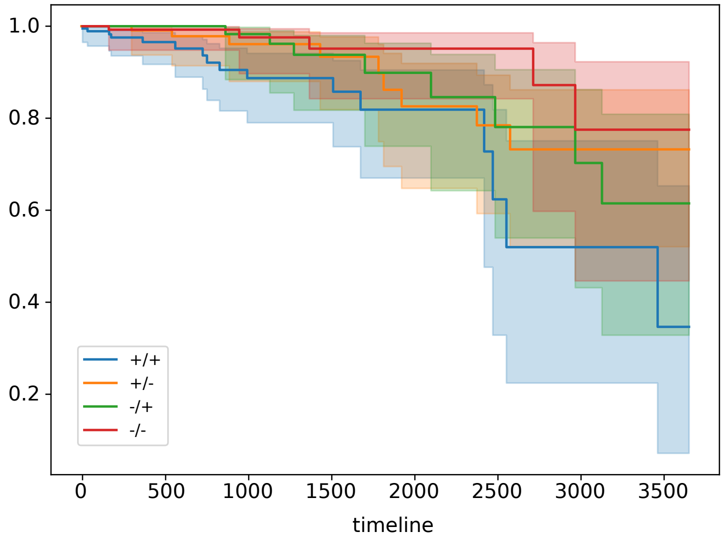

	+/+							
At risk	181	78	50	30	14	6	3	2
Censored	17	116	139	159	173	179	181	181
Events	1	5	10	10	12	14	15	16
	+/-							
At risk	166	82	49	31	22	18	12	6
Censored	9	92	123	140	146	149	154	160
Events	0	1	3	4	7	8	9	9
	-/+							
At risk	169	83	51	29	17	12	8	5
Censored	6	92	123	143	154	157	160	162
Events	0	0	1	3	4	6	7	8
	-/-							
At risk	191	79	54	33	16	13	8	8
Censored	9	120	144	164	181	184	187	187
Events	0	1	2	3	3	3	5	5

Supplementary Figure 6: **Kaplan-Meier plot of patient survival grouped based on both pretreatment proliferation score, and predicted change in score under ET (both binarized at their respective median values).** Note that the change in proliferation score is defined as the difference between predicted post- and pretreament score; because almost all patients' scores decrease, higher values of the change represent smaller such decreases, while lower values indicate larger decreases. The blue curve (labeled +/+) indicates patients with a high score change (small decrease) and high pre-treatment score. They show the worst overall survival. The orange (+/-) and green (-/+) curves indicate patients with high (low) score changes and low (high) pretreatment values. They tend to show intermediate survival. The red curve (-/-) indicates patients with low change (large decrease) and low pretreatment proliferation score. They show the best survival. Survival is shown in days. Width of colored regions surrounding each line indicates 95% confidence interval.

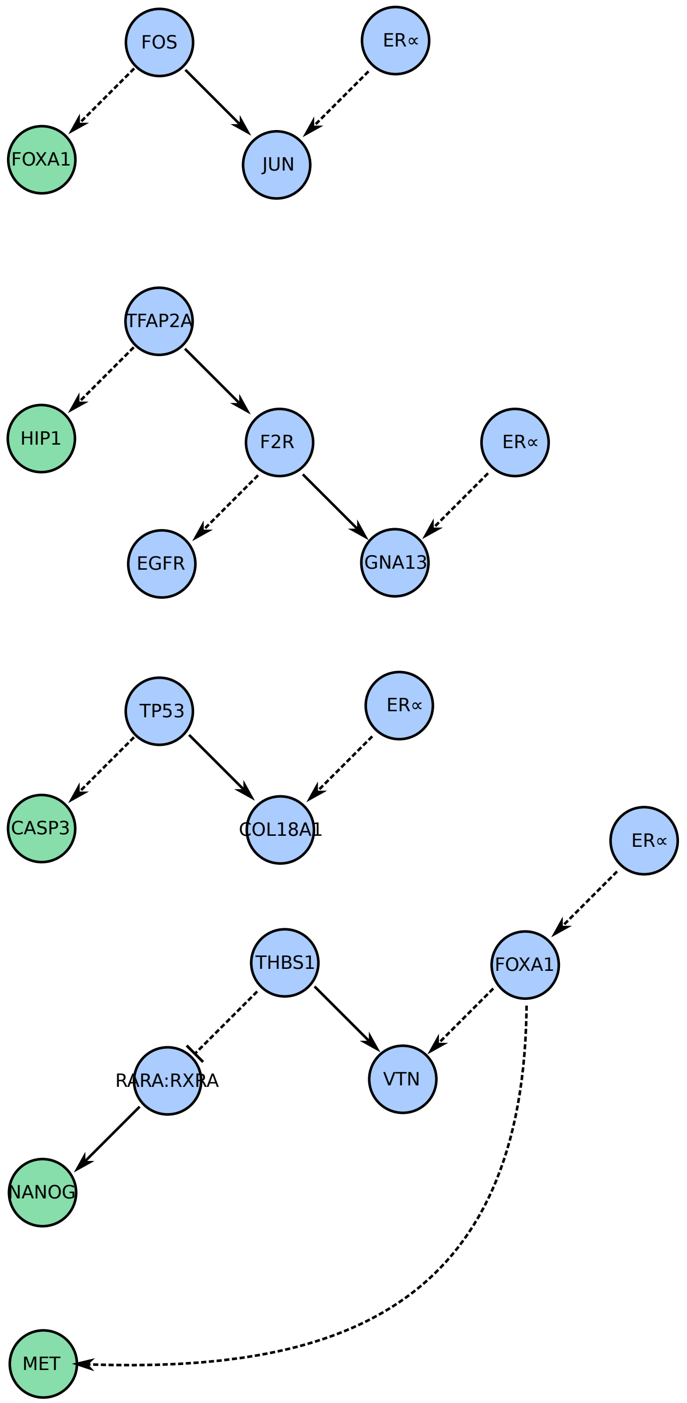

Supplementary Figure 7: **Cartoon indicating how ET is predicted to affect several genes associated with the behavior scores.** For each MDP, the affected gene is shown in green on the left, and the pathway proceeds to the right, terminating at ERα. In all interactions, parent nodes are depicted above child nodes. The pathways regulating *FOXA1*, *HIP1*, and *CASP3* (top three) are all related to the cell death score, while those regulating *NANOG* and *MET* (bottom two) are tightly coupled, and relate to stemness and metastasis, respectively.

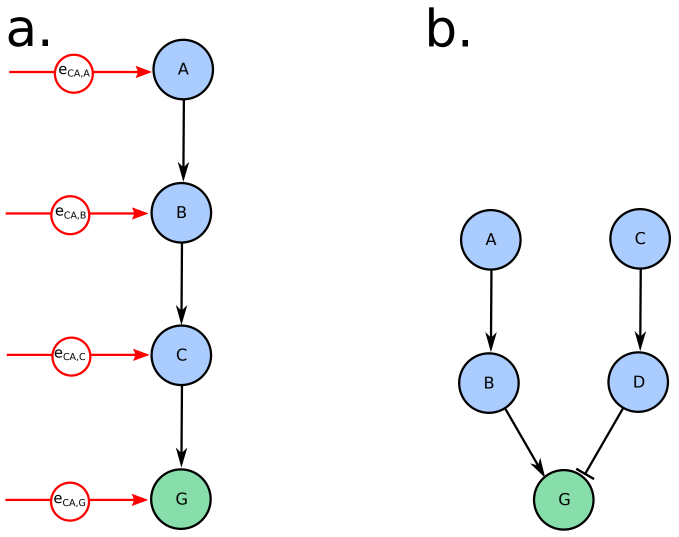

Supplementary Figure 8: **Cartoon indicating how two types of unintended biases arise as a result from the parsimonious objective used in this work.** Panel a. depicts a linear pathway with constitutive activation edges shown. The most parsimonious solution corresponding to some non-zero activation of gene G occurs when the gene is constitutively activated. Barring that possibility, the most parsimonious solution occurs when protein C is constitutively activated, and that activity flows to gene G. In general, the parsimonious solution tends to favor constitutive activation at or near the genes. Panel b. depicts another network motif involving two parallel pathways interacting with gene G. Importantly, the right-hand pathway includes protein D inhibiting gene G. The most parsimonious solution corresponding to some non-zero activation of the gene involves activity flowing through the left-hand pathway; indeed, any activity flowing through the right-hand pathway requires additional activity flowing through the left-hand pathway, rendering such solutions less parsimonious.

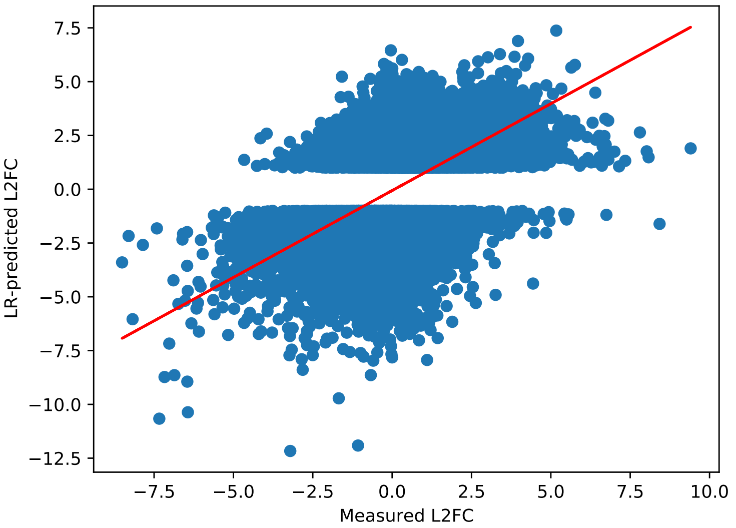

Supplementary Figure 9: **Scatter plot of LR-predicted** *vs.* **measured L2FCs among the** $55,138$ **instances of predicted differential expression in the 25 patients from [11].** Blue dots indicate an instance (some gene in some patient) in which the LR model predicted an absolute L2FC of greater than 1. The red line represents the linear least-squares best fit between predicted and experimental values. Overall, the predictions show a modest correlation with actual L2FC.

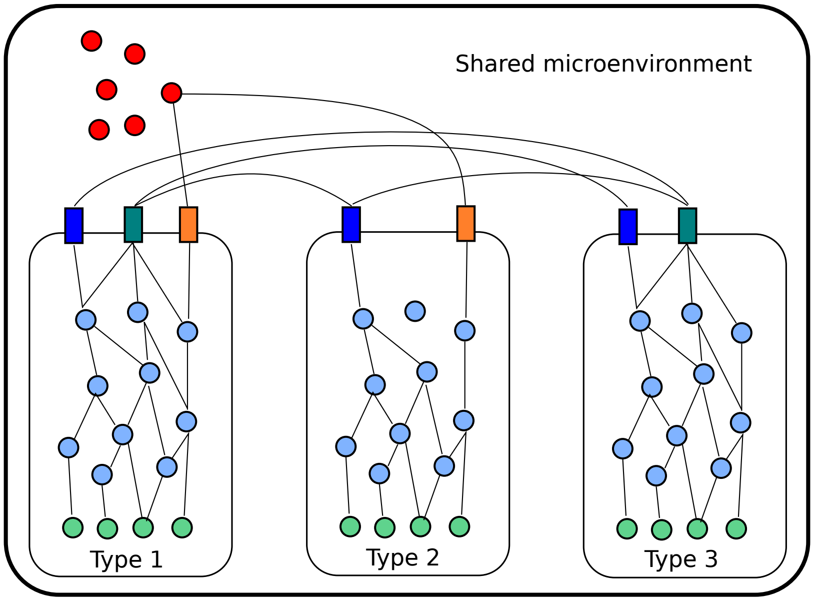

Supplementary Figure 10: **Cartoon representing a possible multiple cell type SCSTA model.** Some bulk RNAseq deconvolution and purification method may be used to construct gene expression estimates for the different cell types present. Each of these would be represented by its own network of intracellular interactions. Secreted molecules (red dots, produced in this case by cell type 1) are able to interact with their downstream targets on the surface of other cell types (to the extent they are expressed, the orange rectangles on cell types 1 and 3), as would other cell surface proteins (*e.g.* blue rectangles interacting with green rectangles). No intracellular proteins can interact with secreted and/or surface-bound proteins other than those associated with their own cell type.

1.11 Supplementary Tables

Score	ER^- mean	ER^+ mean	p-value
proliferation	0.36	-0.11	0.10
cell death	0.75	-0.22	2.1×10^{-4}
cell cycle	1.8	-0.52	1.0×10^{-16}
immune	-1.4	0.41	3.6×10^{-4}
stemness	0.86	-0.25	5.1×10^{-11}
metastasis	-0.29	0.085	1.7×10^{-6}

Supplementary Table 1: **Mean pretreatment behavior scores.** Patients were stratefied by ER-positivity, means were computed among ER-positive and -negative patients, and p-values associated with two-sided Welch's t-tests for differences between the group means were computed

Phenotype	mean ER-negative	mean ER-positive	p-value
M1_polarization	0.22	-0.064	0.00083
T-reg_differentiation	0.23	-0.067	0.00031
Monocyte_differentiation	-0.087	0.026	0.099
Macrophage_activation	0.29	-0.087	9.6e-07
Cytokine_production	0.0	0.0	nan
T_cell_activation	-0.47	0.14	2.6e-20
Basophil_diff	-0.14	0.042	0.021
M2_polarization	-0.26	0.077	2.1e-05
T-lymphocyte_diff	-1.0	0.3	2e-145
Neutrophil_activation	0.0	0.0	nan
B_cell_maturation	0.39	-0.12	3.3e-09
Macrophage_differentiation	-0.31	0.091	0.00015
Mast-Cell_diff	0.055	-0.016	0.37
Neutrophil_degranulation	0.0	0.0	nan
Helper_T-lymphocyte_activation	0.0	0.0	nan
Interferon_Production	0.14	-0.041	0.074
Cytotoxic_T-lymphocyte_activation	0.0	0.0	nan
B-Lymphocyte_diff	-0.95	0.28	2.2e-110
Inflammation	0.03	-0.0088	0.54
Immune_response	0.51	-0.15	3.5e-08

Supplementary Table 2: **The twenty phenotypes comprising the immune score used in this work.** For each, mean values among the ER-negative and ER-positive cohorts, along with p-values from two-sided Welch's t-tests are shown.

Edge	ϕ-value	signed ϕ-value
ERα → *GREB1*	0.13	-0.13
HIF1 → *HK1*	0.1	-0.1
ERα → *AKT1*	0.08	-0.08
E2F1 → *MAPK1*	0.06	-0.06
ERα → *AKT2*	0.056	-0.056
STAT1 → *MAPK1*	0.055	-0.055
NFKB1 → *NQO1*	0.04	-0.04
MYC → *UBTF*	0.032	-0.032
NRF1 → *EIF2S1*	0.031	-0.031
ERα → *NOTCH2*	0.028	-0.028
ERα → *AR*	0.026	-0.026
SP1 → *MAT2B*	0.025	-0.025
TP53 → *HK2*	0.023	-0.023
NFKB1 → *WT1*	0.018	0.018
AR → *PFKFB2*	0.016	-0.016
NFKB1 → *OPTN*	0.016	-0.016
MYC → *MAPK1*	0.015	-0.015
SP1 → *MAPK1*	0.013	-0.013
FOS → *NQO1*	0.012	-0.012
GTF2F1 ⊣ *MYC*	0.011	0.011

Supplementary Table 3: **The twenty edges most-strongly effecting the proliferation score.** In addition to ϕ, the "signed-ϕ", defined identically to that of Equation 11 in the main manuscript but without the absolute value in the numerator, is also given. This later value offers insight into the direction that each edge tends to change the score.

Edge	ϕ-value	signed ϕ-value
ERα → *AKT2*	0.13	0.13
ERα → *AKT1*	0.12	0.12
FOS → *FOXA1*	0.087	−0.087
TFAP2A → *HIP1*	0.082	−0.082
HIF1 → *HK1*	0.077	0.077
TP53 → *CASP3*	0.057	−0.057
ERα:NCOA2 → *BCL2*	0.041	0.041
ERα → *AR*	0.038	0.038
E2F1 → *MAPK1*	0.036	0.036
STAT1 → *MAPK1*	0.033	0.033
ETS1 → *PARP1*	0.027	0.027
NFKB1 → *OPTN*	0.024	0.024
TP53 → *HK2*	0.022	0.022
ERα:NCOA3 → *BCL2*	0.021	0.021
SP1 → *MAT2B*	0.019	0.019
NFIB → *FOXO6*	0.017	−0.017
TP53 → *PML*	0.015	−0.015
TP53 → *HIPK2*	0.014	−0.014
NFY → *TOP2A*	0.013	−0.013
ERα → *FOXO1*	0.0098	−0.0098

Supplementary Table 4: **The twenty edges most-strongly effecting the cell death score.** As above, ϕ, and signed-ϕ are given.

Edge	ϕ-value	signed ϕ-value
ERα → *E2F1*	0.34	−0.34
ERα → *CDKN1A*	0.26	0.26
ERα → *CCND3*	0.15	0.15
AR → *UBE2C*	0.051	0.051
FOXA1 → *BRCA1*	0.047	0.047
STAT1 → *COX5B*	0.03	−0.03
TP53 → *RB1*	0.027	0.027
MYC → *UBE2C*	0.022	0.022
CDK5RAP2 → *BUB1B*	0.019	−0.019
E2F1 → *RB1*	0.015	0.015
NR3C1 → *FOXO3*	0.0094	0.0094
E2F1 → *POLA1*	0.0039	−0.0039
FOS → *FOXO3*	0.0035	0.0035
MEOX2 → *CDKN1A*	0.0034	0.0034
GABPA → *RB1*	0.0032	0.0032
TP53 → *CDKN1B*	0.003	0.003
FOXO4 → *CDKN1B*	0.0026	0.0026
VDR → *CDKN1B*	0.0016	0.0016
GTF2I → *SupplementaryRT1*	0.0016	0.0016
STAT5A → *CCND3*	0.0014	0.0014

Supplementary Table 5: **The twenty edges most-strongly effecting the cell cycle score.** As above, ϕ, and signed-ϕ are given.

Edge	ϕ-value	signed ϕ-value
ERα ⊣ *IL-8*	0.94	0.94
NFKB1 → *TLR7*	0.012	−0.012
TP53 → *TLR8*	0.0089	−0.0089
NFKB1 → *GATA3*	0.0076	−0.0076
STAT1 ⊣ *IL-8*	0.0052	0.0052
E2F1 → *MAPK1*	0.0022	0.0022
STAT1 → *MAPK1*	0.002	0.002
TP63 → *STAT6*	0.0013	−0.0013
TCF7L2 → *STAT3*	0.0009	−0.0009
NFKB1 → *IL-18*	0.00077	−0.00077
CEBPB → *S100A9*	0.00076	0.00076
JUND → *TLR1*	0.00071	−0.00071
CEBPA → *STAT3*	0.00066	−0.00066
STAT1 → *STAT3*	0.00066	−0.00066
GATA3 → *TLR5*	0.00063	−0.00063
NFATC4 → *PTGS2*	0.00058	0.00058
NFKB1 → *TLR2*	0.00057	−0.00057
MYC → *MAPK1*	0.00054	0.00054
STAT3 → *S100A9*	0.00049	0.00049
SP3 → *AGTR1*	0.00048	−0.00048

Supplementary Table 6: **The twenty edges most-strongly effecting the immune score.** As above, ϕ, and signed-ϕ are given.

Edge	ϕ-value	signed ϕ-value
ERα → *CDH1*	0.52	0.52
RARA:RXRA → *NANOG*	0.42	0.42
PAX6 ⊣ *NANOG*	0.015	0.015
ERα → *AR*	0.014	0.014
E2F1 → *MAPK1*	0.0064	0.0064
STAT1 → *MAPK1*	0.0058	0.0058
CEBPA → *SOX4*	0.0045	−0.0045
LEF1 ⊣ *NANOG*	0.004	0.004
PBX1 → *CDH1*	0.0018	0.0018
MYC → *MAPK1*	0.0016	0.0016
SP1 → *MAPK1*	0.0014	0.0014
SPI1 → *ITGAM*	0.0012	0.0012
ERα ⊣ *TFAP2C*	0.001	0.001
NFKB1 → *HIF1A*	0.00096	−0.00096
FOXA1 → *CEBPA*	0.00088	0.00088
TCF7 → *LEF1*	0.00054	0.00054
JUND → *SOX4*	0.0005	−0.0005
STAT1 → *CCR1*	0.00042	0.00042
FOS → *NFATC1*	0.00038	0.00038
NFE2L2 → *PXDN*	0.00034	−0.00034

Supplementary Table 7: **The twenty edges most-strongly effecting the stemness score.** As above, ϕ, and signed-ϕ are given.

Edge	ϕ-value	signed ϕ-value
FOXA1 $\dashv$ *MET*	0.89	0.89
FOS $\rightarrow$ *EZR*	0.033	-0.033
TFAP2A $\rightarrow$ *CRABP2*	0.031	-0.031
FOS $\dashv$ *MET*	0.018	0.018
TFAP2C $\rightarrow$ *CRABP2*	0.0091	-0.0091
TP53 $\dashv$ *MET*	0.0056	0.0056
TP53 $\rightarrow$ *PTTG1*	0.0046	-0.0046
MYH9 $\rightarrow$ *CTNNB1*	0.0017	-0.0017
SupplementaryX1 $\rightarrow$ *EZR*	0.0012	-0.0012
E2F1 $\dashv$ *MET*	0.0011	0.0011
AR $\rightarrow$ *EZR*	0.0011	-0.0011
TCF7L2 $\rightarrow$ *CTNNB1*	0.00099	-0.00099
STAT3 $\rightarrow$ *CTNNB1*	0.00081	-0.00081
SP1 $\rightarrow$ *PTTG1*	0.00017	-0.00017
MYC $\rightarrow$ *NFE2L2*	$1.4e-05$	$-1.4e-05$
CTCF $\rightarrow$ *NFE2L2*	$1e-05$	$-1e-05$

Supplementary Table 8: **All 16 edges that affect the metastasis score.** As above, ϕ, and signed-ϕ are given.

behavior score	SIGNOR phenotype	weight in behavior score
proliferation	Metabolism	1
proliferation	Cell_growth	1
proliferation	Glycolysis	1
proliferation	Protein_synthesis	1
proliferation	Proliferation	1
proliferation	Oxidative_phosphorylation	1
cell death	Survival	-1
cell death	Necroptosis	1
cell death	Apoptosis	1
cell death	Necrosis	1
cell death	Cell_death	1
cell death	Cell_killing	1
cell death	Immortality	-1
cell cycle	Mitotic_checkpoint	1
cell cycle	G2/M_transition	1
cell cycle	Cell_cycle_block	-1
cell cycle	Cell_cycle_exit	-1
cell cycle	G1/S_transition	1
cell cycle	Quiescence	-1
cell cycle	DNA_replication	1
cell cycle	Cell_cycle_progress	1
cell cycle	G1/S_transition_checkpoint	1
cell cycle	G2/M_transition-checkpoint	1
immune	M1_polarization	1
immune	T-reg_differentiation	1
immune	Monocyte_differentiation	1
immune	Macrophage_activation	1
immune	Cytokine_production	1
immune	T_cell_activation	1
immune	Basophil_diff	1
immune	M2_polarization	1
immune	T-lymphocyte_diff	1
immune	Neutrophil_activation	1
immune	B_cell_maturation	1
immune	Macrophage_differentiation	1
immune	Mast-Cell_diff	1
immune	Neutrophil_degranulation	1
immune	Helper_T-lymphocyte_activation	1
immune	Interferon_Production	1
immune	Cytotoxic_T-lymphocyte_activation	1
immune	B-Lymphocyte_diff	1
immune	Inflammation	1
immune	Immune_response	1
stemness	Epithelial-mesenchymal_transition	1
stemness	Differentiation	-1
stemness	Pluripotency	1
metastasis	Metastasis	1

Supplementary Table 9: **SIGNOR phenotypes used in the behavior scores.** In each case, associated score weightings are also given.

References

[1] Marina Parton, Mitchell Dowsett, and Ian Smith. Studies of apoptosis in breast cancer. *Bmj*, 322(7301):1528–1532, 2001.

[2] JA Henry, S Nicholson, JR Farndon, BR Westley, and FEB May. Measurement of oestrogen receptor mrna levels in human breast tumours. *British journal of cancer*, 58(5):600–605, 1988.

[3] Irene K Guttilla, Brian D Adams, and Bruce A White. Erα, micrornas, and the epithelial–mesenchymal transition in breast cancer. *Trends in Endocrinology & Metabolism*, 23(2):73–82, 2012.

[4] Sherene Loi, Nicolas Sirtaine, Fanny Piette, Roberto Salgado, Giuseppe Viale, Françoise Van Eenoo, Ghizlane Rouas, Prudence Francis, JP Crown, Erika Hitre, et al. Prognostic and predictive value of tumor-infiltrating lymphocytes in a phase iii randomized adjuvant breast cancer trial in node-positive breast cancer comparing the addition of docetaxel to doxorubicin with doxorubicin-based chemotherapy: Big 02-98. *J Clin Oncol*, 31(7):860–867, 2013.

[5] Sanaa Al Saleh, Fahd Al Mulla, and Yunus A Luqmani. Estrogen receptor silencing induces epithelial to mesenchymal transition in human breast cancer cells. *PloS one*, 6(6):e20610, 2011.

[6] Christiaan Klijn, Steffen Durinck, Eric W Stawiski, Peter M Haverty, Zhaoshi Jiang, Hanbin Liu, Jeremiah Degenhardt, Oleg Mayba, Florian Gnad, Jinfeng Liu, et al. A comprehensive transcriptional portrait of human cancer cell lines. *Nature biotechnology*, 33(3):306–312, 2015.

[7] Pablo Moreno, Silvie Fexova, Nancy George, Jonathan R Manning, Zhichiao Miao, Suhaib Mohammed, Alfonso Muñoz-Pomer, Anja Fullgrabe, Yalan Bi, Natassja Bush, et al. Expression atlas update: gene and protein expression in multiple species. *Nucleic acids research*, 50(D1):D129–D140, 2022.

[8] Zhi-Jian Han, Yang-Bing Li, Lu-Xi Yang, Hui-Juan Cheng, Xin Liu, and Hao Chen. Roles of the cxcl8-cxcr1/2 axis in the tumor microenvironment and immunotherapy. *Molecules*, 27(1):137, 2021.

[9] Wai Leong Tam, Haihui Lu, Joyce Buikhuisen, Boon Seng Soh, Elgene Lim, Ferenc Reinhardt, Zhenhua Jeremy Wu, Jordan A Krall, Brian Bierie, Wenjun Guo, et al. Protein kinase c α is a central signaling node and therapeutic target for breast cancer stem cells. *Cancer cell*, 24(3):347–364, 2013.

[10] Robert Clarke, Brandon C Jones, Catherine M Sevigny, Leena A Hilakivi-Clarke, and Surojeet Sengupta. Experimental models of endocrine responsive breast cancer: Strengths, limitations, and use. *Cancer Drug Resistance*, 4(4):762, 2021.

[11] Youli Xia, Xiaping He, Lorna Renshaw, Carlos Martinez-Perez, Charlene Kay, Mark Gray, James Meehan, Joel S Parker, Charles M Perou, Lisa A Carey, et al. Integrated dna and rna sequencing reveals drivers of endocrine resistance in estrogen receptor–positive breast cancer. *Clinical Cancer Research*, 28(16):3618–3629, 2022.

[12] Adriana Papadimitropoulou, Luciano Vellon, Ella Atlas, Travis Vander Steen, Elisabet Cuyàs, Sara Verdura, Ingrid Espinoza, Javier A Menendez, and Ruth Lupu. Heregulin drives endocrine resistance by altering il-8 expression in er-positive breast cancer. *International journal of molecular sciences*, 21(20):7737, 2020.

[13] Nataša Todorović-Raković and Jelena Milovanović. Interleukin-8 in breast cancer progression. *Journal of Interferon & Cytokine Research*, 33(10):563–570, 2013.

[14] Xiaoyong Fu, Rinath Jeselsohn, Resel Pereira, Emporia F Hollingsworth, Chad J Creighton, Fugen Li, Martin Shea, Agostina Nardone, Carmine De Angelis, Laura M Heiser, et al. Foxa1 overexpression mediates endocrine resistance by altering the er transcriptome and il-8 expression in er-positive breast cancer. *Proceedings of the National Academy of Sciences*, 113(43):E6600–E6609, 2016.

[15] Yu Jin Kim, Jong-Sun Choi, Jinwon Seo, Ji-Young Song, Seung Eun Lee, Mi Jung Kwon, Mi Jeong Kwon, Juthika Kundu, Kyungsoo Jung, Ensel Oh, et al. Met is a potential target for use in combination therapy with egfr inhibition in triple-negative/basal-like breast cancer. *International journal of cancer*, 134(10):2424–2436, 2014.

[16] Yebin Im and Yongsoo Kim. A comprehensive overview of rna deconvolution methods and their application. *Molecules and cells*, 46(2):99–105, 2023.

[17] Lihua Jiang, Meng Wang, Shin Lin, Ruiqi Jian, Xiao Li, Joanne Chan, Guanlan Dong, Huaying Fang, Aaron E Robinson, François Aguet, et al. A quantitative proteome map of the human body. *Cell*, 183(1):269–283, 2020.
